# Satellite Glial Cells Synthesize and Release GABA to Activate Extrasynaptic GABA_A_ Receptors That Modulate Dorsal Root Ganglia Neuron Excitability

**DOI:** 10.1002/glia.70190

**Published:** 2026-07-20

**Authors:** Natalie Jiménez‐Barrios, Ricardo González‐Ramírez, Francisco Javier Paz‐Bermúdez, Guadalupe Raya‐Tafolla, José Segovia Vila, Ricardo Felix, Rodolfo Delgado‐Lezama, Benjamín Florán Garduño

**Affiliations:** ^1^ Department of Physiology, Biophysics and Neurosciences Center for Research and Advanced Studies of the National Polytechnic Institute Mexico City Mexico; ^2^ Department of Molecular Biology and Histocompatibility “Dr. Manuel Gea González” General Hospital Mexico City Mexico; ^3^ Center for Research on Aging, Center for Research and Advanced Studies of the National Polytechnic Institute Mexico City Mexico; ^4^ Department of Cell Biology Center for Research and Advanced Studies of the National Polytechnic Institute Mexico City Mexico

**Keywords:** best 1, DRG, GABA release, GABA_A_ receptors, satellite glia

## Abstract

Sensory neurons express extrasynaptic GABA_A_ receptors in their soma and axon, tonically activated by ambient GABA, modulating their excitability. However, the specific glial or neuronal origin of endogenous GABA that modulates this excitability has yet to be identified. We investigated the expression and function of enzymes involved in GABA synthesis via the ornithine‐putrescine and glutamic acid pathways, and the effects of inhibiting these enzymes on the compound action potential (cAP) of primary afferent fibers. PCR analysis revealed that the dorsal root ganglia (DRG) express transcripts for ornithine decarboxylase (ODC), monoamine oxidase B (MAOB), diamine oxidase (DAO), and GAD65/67. Immunofluorescence assays confirmed the expression of ODC, MAOB, DAO, and GAT‐3 proteins, as well as GABA in satellite glial cells (SGC). In contrast, neurons express DAO and ODC. However, despite the presence of GAD65 and GAD67 mRNAs, their corresponding proteins were not detected. Inhibition of ODC and MAOB, but not DAO or GAD, prevented the accumulation of GABA induced by the GABA transaminase (GABA‐T) inhibitor aminooxy acetic acid in SGC cultures. Additionally, the Best1 channel blocker CaCCinh suppressed the K^+^‐induced release of [^3^H]GABA in DRG and SGC cultures. Blocking GABA_A_ receptors with picrotoxin, inhibiting MAOB, and blocking Best1 all increased cAP. However, allylglycine, a GAD inhibitor, failed to elicit this effect. Likewise, the use of selegiline and CaCCinh on cAP occluded the effects of picrotoxin. These results support that GABA synthesized and released by satellite glial cells activates extrasynaptic GABA_A_ receptors, thereby modulating the excitability of sensory neurons.

## Graphical abstract

Mechanism of synthesis, release, receptor activation, uptake, and degradation of GABA in dorsal root ganglia (DRG). Left: GABA is synthesized from putrescine in satellite glial cells (SGCs). After being released by Best1 channel, it activates GABA_A_ receptors in sensory neurons, producing a chloride current. This GABA is removed from the extracellular space by GAT3 and degraded by GABA‐T. In these conditions, sensory neurons produce a normal amplitude of compound action potential (cAP). Right: Blockade of the MAOB or Best1 channel caused a reduction in chloride current. These increases in membrane resistance of sensory neurons increase the amplitude of cAP.

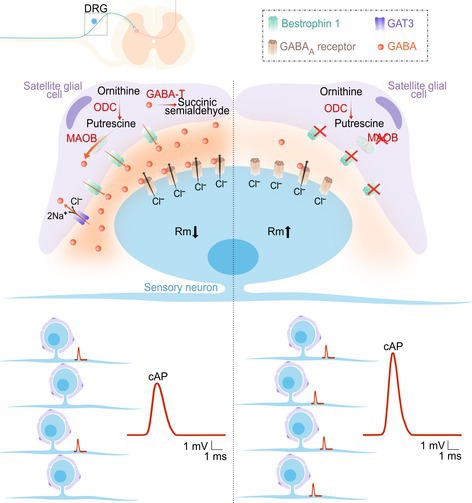

## 1 Introduction

Sensory neurons transmit information from the periphery to the central nervous system (CNS). These neurons express extrasynaptic GABA_A_ *α*_5/6_ receptors along their axons and within their soma, which are located in the dorsal root ganglia (DRG) in mammals (Bravo‐Hernández et al. 2016; Loeza‐Alcocer et al. 2013; Rodríguez‐Palma et al. 2023; Perez‐Sanchez et al. 2017). Tonic activation of these receptors is necessary to regulate the excitability of sensory neurons (Hernández‐Reyes et al. 2019; Loeza‐Alcocer et al. 2013).

This modulation is significant because pathological pain states, such as allodynia and hyperalgesia, have been linked to dysregulated excitability of DRG neuronal somas and axons (Pineda‐Farias et al. 2015). However, the source of the GABA that activates these extrasynaptic receptors remains unknown, raising a key research question: which DGR cell type is responsible for GABA production? While the primary enzyme in the central nervous system (CNS) that produces GABA from glutamate is glutamic acid decarboxylase (GAD), astrocytes can also use the polyamine pathway to produce GABA from ornithine and putrescine (Watanabe et al. 2002; Yoon et al. 2014). Nevertheless, the identification of the GABA‐producing cell type in the DRG remains a topic of debate.

According to recent data, GABA released from astrocytes, not GABAergic interneurons, mediates the tonic activation of extrasynaptic GABA_A_ receptors (Ju et al. 2024). This indicates that the interneurons responsible for activating synaptic GABA_A_ receptors and mediating primary afferent depolarization at the central terminals of sensory neurons (Witschi et al. 2011) are not the source of ambient GABA in the DRG.

Building on these findings on the DRG, autoradiographic studies have demonstrated that [^3^H]GABA uptake was localized exclusively in satellite glial cells while the neuronal cell bodies, remnants of the connective tissue sheath, and the myelinated fibers lack labeling (Beart et al. 1974; Gottesfeld et al. 1973; Schon and Kelly 1974b). Furthermore, earlier studies indicated that GABA can be released through the Bestrophin 1 (Best1) channel in the brain (Joo et al. 2024; Lee et al. 2010). In the turtle DRG, depolarization‐induced [^3^H]GABA release is extracellular Ca^2+^‐independent and sensitive to Best1 blocker NPPB, indicating the possible glial source of GABA (Vargas‐Parada et al. 2021).

Recently, it was demonstrated that astrocyte‐specific knockout of Best1 reduces GABAergic tonic inhibition, which affects neuronal excitability in the rat brain (Joo et al. 2024). Additionally, it has been suggested that the somas of sensory neurons in the DRG can synthesize GABA using the enzyme GAD67 and release it through a vesicle‐dependent pathway; however, there is currently no concrete proof of functional GABA release (Du et al. 2017). The identity of the cell type that produces and releases GABA—with the ability to activate extrasynaptic GABA_A_ receptors and modulate the excitability of DRG neurons—remains controversial, as both satellite glial cells (SGC) and neurons work together as a functional unit in both healthy and diseased states (Hanani 2005). Given their similarities, it is possible that SGC and astrocytes could synthesize and release GABA similarly, though this has not been thoroughly studied (Hanani and Verkhratsky 2021).

Therefore, the purpose of this study was to identify the cellular source, the DRG's GABA synthesis and release mechanisms, and how these processes control the excitability of sensory neurons. Our data, which were obtained using molecular biology, neurochemistry, and electrophysiology techniques, show that SGC can modulate the excitability of DRG neurons by synthesizing GABA via the ornithine–putrescine pathway, releasing it through the Best1 channel, and activating extrasynaptic GABA_A_ receptors.

## 2 Methods

### 2.1 Animals

Male Wistar rats (180–220 g) were maintained in a controlled environment at 22°C, under a 12 h light/dark cycle with food and water *ad libitum*. All procedures followed the National Institutes of Health Guide for Care and Use of Laboratory Animals and were approved by the Institutional Animal Care Committee of the CINVESTAV, making all efforts to minimize animal suffering (Protocol 0198‐16, Center for Research and Advanced Studies, Mexico City, Mexico), which complied with the Official Mexican Standard (NOM‐062‐ZOO‐1999).

### 2.2 Dorsal Root Ganglion Neuron Isolation and Culture

Animals were sacrificed by decapitation. The vertebral column was exposed by cutting the skin, muscles, and ribs. The spinal cord was immediately extracted by mechanical extrusion. According to the experiment, the vertebral apophysis was cut to expose and remove all lumbar DRG for further processing.

SGC cultures were prepared using previously published protocols (Bustamante et al. 2021; Capuano et al. 2009). Dorsal and ventral roots were carefully removed from DRG following sterile harvesting, and the ganglia were dissected in ice‐cold phosphate‐buffered saline (PBS). The DRG were then enzymatically dissociated at 37°C for 40 min using 2.5 mg/mL collagenase/papain in PBS. Following removal of the enzymatic solution, the tissue was mechanically homogenized and resuspended in F12/DMEM medium. The cell suspension was then centrifuged at 5000 rpm for 4 min, after which the pellet was resuspended in F12/DMEM containing 1% penicillin/streptomycin and 10% fetal bovine serum.

Cells were plated in T75 flasks with 6 mL of supplemented medium and incubated at 37°C in a humidified atmosphere containing 5% CO_2_. After 4 h, the medium was replaced to remove unattached cells. Cultures were maintained until confluence (10–14 days), after which cells were trypsinized and replated as required for subsequent experiments.

### 2.3 RT‐PCR

Total RNA was extracted from bilateral L1–L6 DRG using TRIzol Reagent. To put it briefly, RNA was extracted using chloroform and then centrifuged at 12,000 g for 15 min at 4°C, as directed by the manufacturer (Invitrogen Life Technologies). The pellet was washed in 70% ethanol and suspended in diethyl pyrocarbonate‐treated water. The total RNA concentration was determined by spectrometric analysis with an Epoch Microplate Spectrophotometer (BioTek). Single‐strand cDNA was synthesized from the extracted RNA (5 μg) with M‐MLV reverse transcriptase (Invitrogen) and oligo‐dT (50 pmol), then 50 μL of the resulting cDNA was used for PCR. The sequences of the oligonucleotides used for PCR amplification were designed in VectorNTI software (Table S1). A thermocycler (Thermo Fisher Scientific) was used for PCR, and 40 cycles of 94°C for 45 s, 55°C for 30 s, and 72°C for 1 min comprised the cDNA amplification in a 50 μL total volume. A BioDoc‐It System (UVP) was used to record images and electrophorese the PCR results in 2% agarose gels stained with ethidium bromide.

### 2.4 Immunofluorescence

Isolated DRG were fixed in 4% PFA in PBS for 48 h and then in 30% saccharose in PBS for 24 h. DRG slices of 20 μm were cut using a cryostat (CM1520, Leica). Sections were washed three times with PBS for 5 min and then permeabilized with 0.3% Triton X‐100 in PBS buffer (PBS‐T) for 10 min. After slices were suspended in SDS 1% in PBS for 5 min, followed by one wash with PBS. Sections were blocked with 2% Bovine Serum Albumin (BSA) IgG‐Free, Protease‐Free with 5% Tween20 in PBS for 2 h at room temperature. Primary antibodies were diluted in 0.2% Triton X‐100 with 1% BSA in PBS and incubated overnight at 4°C. See Table S2 for antibody information. Slices were incubated with a secondary antibody for 2 h at room temperature. Finally, DRG sections were incubated with Hoechst nucleic acid stain. DRG sections were mounted in microscope slices with VECTASHIELD Antifade Mounting Medium. Staining was visualized using epifluorescence microscopy (BA410E, Motic), and images were taken with Image‐Pro Premier software.

### 2.5 Western Blot

Protein extraction from DRG was obtained with RIPA buffer containing (in mM) 150 mM NaCl, 50 Tris [pH 8.0], with 1% NP‐40, 0.5% sodium deoxycholate, 0.1% SDS, 0.5 PMSF, and Complete 1X. The protein used for the vGAT antibody was extracted with NP40 buffer containing (in mM) 150 mM sodium chloride, 1% NP‐40, and 50 mM Tris, pH 8.0. Proteins (35 μg) in Laemmli 1X buffer (1.6% SDS, 0.1 M 2%–5% glycerol, 0.083 M 4X Tris‐HCl/SDS pH 6.8 and 0.002% bromophenol blue) were heated at 100°C for 5 min (except the protein used for the vGAT antibody), separated by SDS‐PAGE and transferred to 0.45 μm nitrocellulose membranes for 1.5 h at ∼18 V. Membranes were blocked with 5% nonfat milk in TBST buffer for 2 h at room temperature and incubated overnight at 4°C. See Table S2 for antibody information. Then, membranes were incubated with anti‐mouse or anti‐rabbit horseradish peroxidase (HRP)‐coupled secondary antibodies, revealed by a chemiluminescence detection system (Thermo Scientific), and were visualized with the Odyssey Fc Imaging System (LI‐COR).

### 2.6 GABA Quantification

SGC were plated at a density of 4.5 × 10^5^ cells per 60 mm culture dish pretreated with 0.05% poly‐L‐lysine. Cells were split into four treatment groups for each experiment: (1) untreated control; (2) treatment with 200 μM aminooxy acetic acid (AOAA) to inhibit GABA transaminase (GABA‐T) and promote intracellular GABA accumulation; (3) treatment with either selegiline (500 nM), berenil (200 μM), allylglycine (5 mM), or eflornithine (10 mM) all of which act as MAOB, DAO, GAD, or ODC inhibitors respectively; or (4) co‐treatment with AOAA and one of the agents mentioned in group 3. All treatments were performed in PBS for 1 h at 37°C. Following incubation, cells were detached using trypsin–EDTA and centrifuged at 5000 rpm for 5 min. Pellets were resuspended in RIPA buffer and maintained on ice for 20 min, followed by a second centrifugation step. The resulting supernatants were analyzed for GABA content via high‐performance liquid chromatography (HPLC) with fluorometric detection (ECD; Intro, Antec Leyden). GABA content was measured by pre‐column derivatization with O‐phthalaldehyde (OPA), and 35 μL of each sample was filtered through a 0.45 μm nylon membrane and injected into the system. Detection was conducted using a glassy carbon electrode (VT‐03; Antec Leyden) set at −550 mV relative to an Ag/AgCl reference electrode (Quiróz‐González et al. 2013).

The Bradford assay was used to determine each sample's total protein concentration. GABA levels were expressed as a percentage of the control values after being normalized to the protein content (ng/μg protein).

### 2.7 [ ^3^H]GABA Release

DRG were maintained in cold and oxygenated Krebs–Henseleit buffer (in mM: 118.25 NaCl, 1.75 KCl, 1 MgSO_4_, 1.25 KH_2_PO_4_, 25 NaHCO_3_, 2 CaCl_2_, and 10 sucrose), continuously bubbled with a 95% O_2_/5% CO_2_ gas mixture. The tissues were then transferred to 2 mL of Krebs–Henseleit buffer at 37°C and incubated for 30 min to allow temperature equilibration. Subsequently, DRG were incubated with [^3^H]GABA (100 nM) for 30 min at 37°C in the presence of aminooxy acetic acid (AOAA, 10 μM) to prevent GABA degradation by GABA‐T. After labeling, tissues were placed in chambers of a continuous perfusion system (0.5 mL/min), as previously described (Floran et al. 2002). Before fraction collection, samples were perfused for 40 min with Krebs–Henseleit buffer supplemented with AOAA and nipecotic acid (10 μM), a GABA uptake inhibitor, to prevent extracellular GABA reuptake (designated as Krebs‐AOAA+NA).

To evaluate the effects of pharmacological agents on [^3^H]GABA release, tissue chambers were divided into four experimental groups: (1) basal release, (2) high K^+^‐induced release, (3) basal release with inhibitors, and (4) high K^+^‐induced release with inhibitors. Thirty fractions were collected at 4‐min intervals to assess basal release, during which tissue samples were continuously perfused with Krebs‐AOAA+NA solution (groups 1 and 3). In groups 2 and 4, a high K^+^ solution (80 mM) was applied during fraction 5 (at 20 min) and removed at fraction 20 to induce depolarization‐dependent release. Following depolarization, ten additional fractions were collected in the presence of standard Krebs‐AOAA+NA solution (without high K^+^) to assess reversibility, as previously described (Floran et al. 2002; Minchin and Iversen 1974).

The composition of the high K^+^ solution was (in mM): 41.25 NaCl, 78.75 KCl, 1 MgSO_4_, 1.25 KH_2_PO_4_, 25 NaHCO_3_, 0.01 AOAA, 2 CaCl_2_, and 10 sucrose.

Experiments were repeated using standard and high K^+^ solutions, substituting Ca^2+^ for Mg^2+^, in order to assess the dependence of [^3^H]GABA release on extracellular calcium. To assess the involvement of voltage‐gated calcium channels, nifedipine (10 μM), ω‐Agatoxin (200 nM), and ω‐Conotoxin (300 nM) were added to the perfusion medium. To evaluate whether [^3^H]GABA release depends on calcium inside cells, tissue samples were first treated with the Ca^2+^ chelator BAPTA‐AM (500 μM) in buffer. The possible role of Best1 channels was also checked by adding the chloride channel blockers NPPB (200 μM) and CaCCinh‐A01 (200 μM).

In all experiments, basal release was subtracted point by point from the induced release. Total [^3^H]GABA release was determined by resuspending the collected fractions in scintillation fluid. The DRG tissues were incubated in 1 N HCl for 1 day, after which scintillation fluid was added. Radioactivity was quantified as previously described (Floran et al. 2002), and baseline values were subtracted for statistical analysis. Fractional [^3^H]GABA release was initially expressed as a proportion of the total tritium remaining in the tissue. Treatment effects were evaluated by calculating the area under the curve (AUC) after subtracting the corresponding basal GABA release for statistical analysis.

[^3^H]GABA release in SGC cultures. SGC cultures were incubated with 80 mM [^3^H]GABA for 30 min in Krebs‐AOAA solution. The label was removed, and the cultures were washed 3 times with Krebs‐AOAA+NA. Next, they were incubated for 15 min in Krebs‐AOAA+NA (80 mM K^+^) to induce depolarization‐induced release. The supernatant was collected in scintillation fluid. The SGC in the dish were lysed in 1 N HCl overnight, after which scintillation fluid was added. Radioactivity was determined by scintillation counting, and data expressed as the fractional release: amount of radioactivity in the supernatant divided by the total radioactive uptake.

### 2.8 Recording of Compound Action Potential (cAP)

DRG were obtained from anesthetized rats by laminectomy, preserving continuity with spinal nerves and dorsal roots. Tissues were immediately placed in ice‐cold, oxygenated Krebs–Henseleit solution containing (in mM): 140 NaCl, 4 KCl, 12 NaHCO_3_, 1 MgCl_2_, 11 glucose, 2 CaCl_2_, and 10 sucrose. The solution was continuously bubbled with 95% O_2_ and 5% CO_2_ and maintained at room temperature (~22°C).

The tissue was transferred into a recording chamber perfused with Krebs solution in order to conduct electrophysiological recordings. Dorsal roots and spinal nerves were placed into a glass suction electrode that was connected to an AC amplifier and a stimulator that delivered rectangular pulses, and dorsal roots were similarly placed into a suction electrode connected to an AC amplifier.

Compound action potential (cAP) was evoked by stimulation of the spinal nerve using 200 μs pulses delivered every 3 s. cAP was recorded from the dorsal root using an amplifier with a gain of X10^3^ and a bandwidth of 0.1–3 kHz. One hundred traces were digitized at a frequency of 40 kHz, averaged, and then analyzed using pClamp software (Molecular Devices). The smallest stimulus current required to produce a cAP 50% of the time was known as the cAP threshold (T). Recordings were then made at twice this threshold (2 × T) to activate low‐threshold primary afferent fibers (A*β* fibers) (Vargas‐Parada et al. 2021).

cAP was measured in the presence of picrotoxin (100 μM), a GABA_A_ receptor antagonist, in order to evaluate the contribution of GABA_A_ receptor activity to afferent excitability. Additional pharmacological manipulations included incubation with selegiline (500 nM), berenil (200 μM), allylglycine (5 mM), CaCCinh (50 μM), and combinations with picrotoxin. All drugs were incubated until stabilization of cAP recordings (2–3 h). cAP responses were quantified as the area under the curve (AUC) across experimental conditions.

### 2.9 Determination of GABA Content in DRG

DRG obtained as previously described were incubated with oxygenated Krebs–Henseleit solution containing selegiline (500 nM) or allylglycine (5 mM) for 1, 2, or 3 h. At the end of this period, ganglia were homogenized in 100 μL and centrifuged at 13800 rpm for 8 min. Pellets were resuspended in NaOH 0.1 N 200 μL, and 2 μL were used for protein determination using Bradford's method. 35 mL of the resulting supernatants were analyzed for GABA content via high‐performance liquid chromatography (HPLC) with electrochemical detection by OPA methods as previously described (Quiróz‐González et al. 2013).

### 2.10 Statistical Analysis

Data were graphed as Tukey boxes and were analyzed using nonparametric methods due to small sample sizes and non‐Gaussian distributions. GABA accumulation experiments and GABA release in SGC cultures were analyzed using the Kruskal‐Wallis test followed by Dunn's test; for GABA release in DRG, we used the Mann–Whitney test; and for cAP data, we used the Friedman test followed by Dunn's test. A *p* < 0.05 was considered statistically significant.

### 2.11 Drugs

5‐nitro‐2‐(3‐phenylpropylamino) benzoic acid (N4779), NPPB; 6‐(1,1‐Dimethylethyl)‐2‐[(2‐furanylcarbonyl)amino]‐4,5,6,7‐tetrahydro‐benzo[b]thiophene‐3‐carboxylic acid (SML0916), CaCCinh‐A01; Collagenase from *Clostridium histolyticum* (C9891); Diminazene aceturate (D7770), Berenil; O‐(Carboxymethyl)hydroxylamine hemihydrochloride (C13408), AOAA; Papain from *papaya latex* (P4762); Picrotoxin (P1675); R‐(−)‐Deprenyl hydrochloride (PHR3134), Selegiline; R(−)‐Nipecotic acid (211672), Nipecotic acid; 1,4‐Dihydro‐2,6‐dimethyl‐4‐(2‐nitrophenyl)‐3,5‐pyridinedicarboxylic acid dimethyl ester, Nifedipine (N7634) were purchased from Sigma‐Aldrich St. Louis, MO, USA. F12/DMEM medium (12634010); Fetal Bovine Serum (16000044); Penicillin/streptomycin (1037801) from Gibco Life Technologies Inc., Grand Island, NY, USA. Bovine Serum Albumin IgG‐Free, Protease‐Free (001‐000‐162), BSA, from Jackson ImmunoResearch, West Grove, PA, USA. VECTASHIELD Antifade Mounting Medium (H‐1000), from Vector Laboratories, Burlingame, CA, USA. L‐Allylglycine (sc‐255,236), from Santa Cruz Biotechnology, Dallas, TX, USA. 1,2‐Bis(2‐aminophenoxy)ethane‐N, N, N′, N′‐tetra acetic acid tetrakis (acetoxymethyl ester) (B6769), BAPTA‐AM; Chemiluminescence detection system Moloney Murine Leukemia Virus Reverse Transcriptase (28025013), M‐MLV RT; TRIzol (15596026) from Thermo Scientific, Waltham, MA, USA. *ω*‐agatoxin‐Aa4a (A‐500), *ω*‐Agatoxin‐TK was obtained from Alomone Labs, Jerusalem, Israel. *ω*‐conotoxin‐GVIA (343781) was purchased from Merck Millipore, Darmstadt, Germany.

Radiochemicals: Aminobutyric Acid (GABA) *γ*‐[2,3‐^3^H(N)]‐GABA, Specific Activity: 70.0 Ci/mmol (2.59TBq/mmol), 1 mCi (37 MBq) (NET191x001MC), [^3^H] GABA was purchased from Perkin Elmer, Springfield, IL, USA.

## 3 Results

### 3.1 The Enzymes From the Ornithine–Putrescine Pathway, but Not From the Glutamic Acid Pathway, Are Present in SGC and Play a Role in GABA Synthesis

**FIGURE 1:**
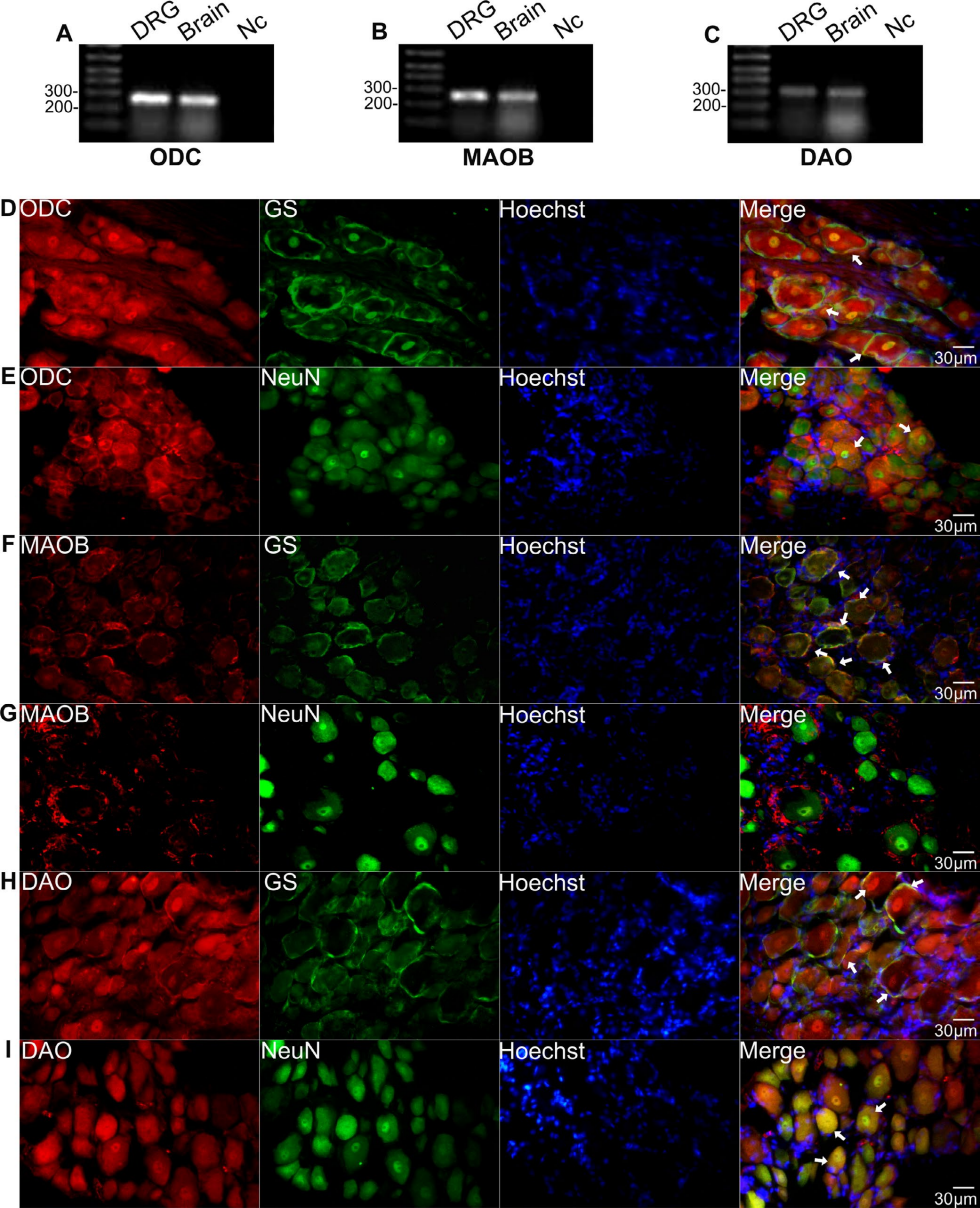
Expression of the ornithine‐putrescine enzyme pathway for GABA synthesis in the DRG. Panels (A–C) display a representative blot from the PCR analysis of ornithine decarboxylase (ODC), monoamine oxidase B (MAOB), and diamine oxidase (DAO) transcripts obtained from the whole DRG mRNA. All transcripts are expressed in the DRG, with whole‐brain mRNA used as a positive control and without RNA as a negative control (Nc). Molecular weight markers are on the left. Panels (D, E) illustrate the immunohistochemical co‐localization of ODC in glutamate synthase (GS)‐positive cells, specifically satellite glial cells panel (D), and in NeuN‐positive neurons panel (E). Panels (F, G) show the co‐localization of MAOB in GS‐positive cells but not in NeuN‐positive elements. Finally, panels (H, I) show the co‐localization of DAO in both cell types. In all cases, arrows indicate colocalization sites.

First, to determine the cellular source of GABA that modulates DRG neuron excitability, we analyzed the expression of key enzymes involved in the glutamic acid and ornithine–putrescine pathways of GABA synthesis in DRG cells using RT‐PCR and immunofluorescence. Figure 1A–C show RT‐PCR detection of transcripts for ornithine decarboxylase (ODC), monoamine oxidase B (MAOB), and diamine oxidase (DAO) in total mRNA extracted from rat DRG tissue. All three transcripts were detected, with their expression verified in whole‐brain samples, which served as positive controls. We also performed immunofluorescence assays to identify the specific cell types expressing these enzymes (Figure 1D–I). The results of this analysis show that SGC, identified by glutamine synthetase (GS) immunoreactivity, co‐expressed ODC (Figure 1D), MAOB (Figure 1F), and DAO (Figure 1H). In contrast, DRG neurons, labeled with the neuronal marker NeuN, co‐expressed DAO (Figure 1E) and ODC (Figure 1I) but not MAOB (Figure 1G).

**FIGURE 2:**
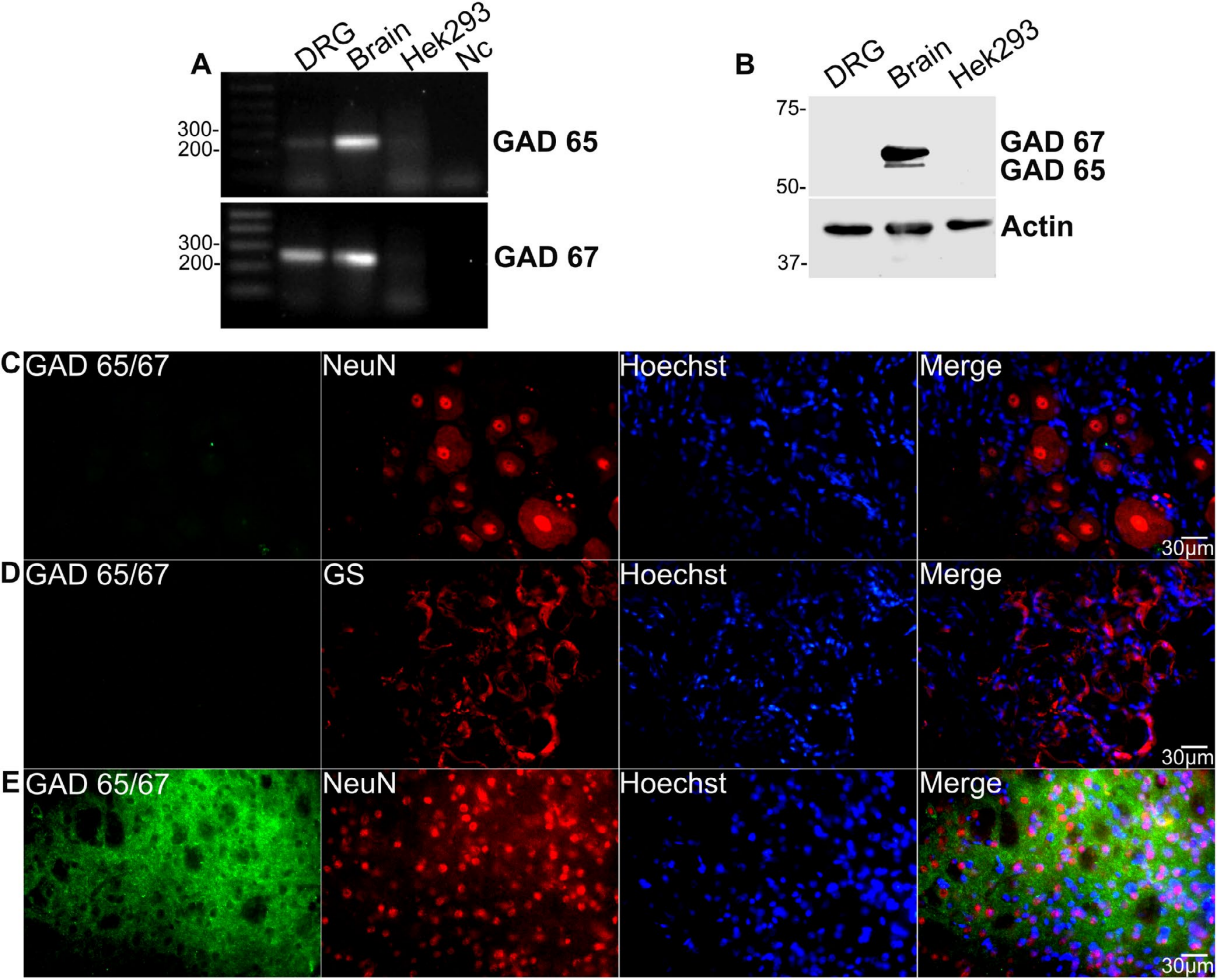
Expression of glutamic acid pathway enzymes involved in GABA synthesis in the DRG. Panel (A) displays a representative blot showing the PCR results for GAD65 and GAD67 transcripts in DRG mRNA. Molecular weight markers are on the left. Panel (B) presents the Western blot results for GAD65/67 from DRG homogenates, with brain tissue as a positive control. Panels (C, D) illustrate immunofluorescence images for glutamine synthetase (GS) and NeuN in the DRG, noting the absence of staining with the GAD65/67 antibody. Lastly, panel (E) shows a positive control from striatal tissue, highlighting the co‐localization of the GAD65/67 antibody with NeuN‐positive elements.

Figure 2 shows RT‐PCR detection of GAD65 and GAD67 transcripts, key enzymes in GABA synthesis, in total mRNA from DRG tissue (Figure 2A). Western blot analysis of DRG homogenates (Figure 2B) did not, however, show detectable protein expression of either isoform, even though the transcripts were present. As positive controls, brain homogenates displayed protein bands for GAD65 and GAD67, and a negative control homogenate from HEK293 cells that do not express enzymes. Immunofluorescence analysis provided additional support for these findings. Neither GAD65 nor GAD67 immunoreactivity was observed in DRG sections, including NeuN‐positive neurons (Figure 2C) or glutamine synthetase‐positive SGC (Figure 2D). In this series of experiments, GAD immunostaining in the rat striatum served as a positive control (Figure 2E).

**FIGURE 3:**
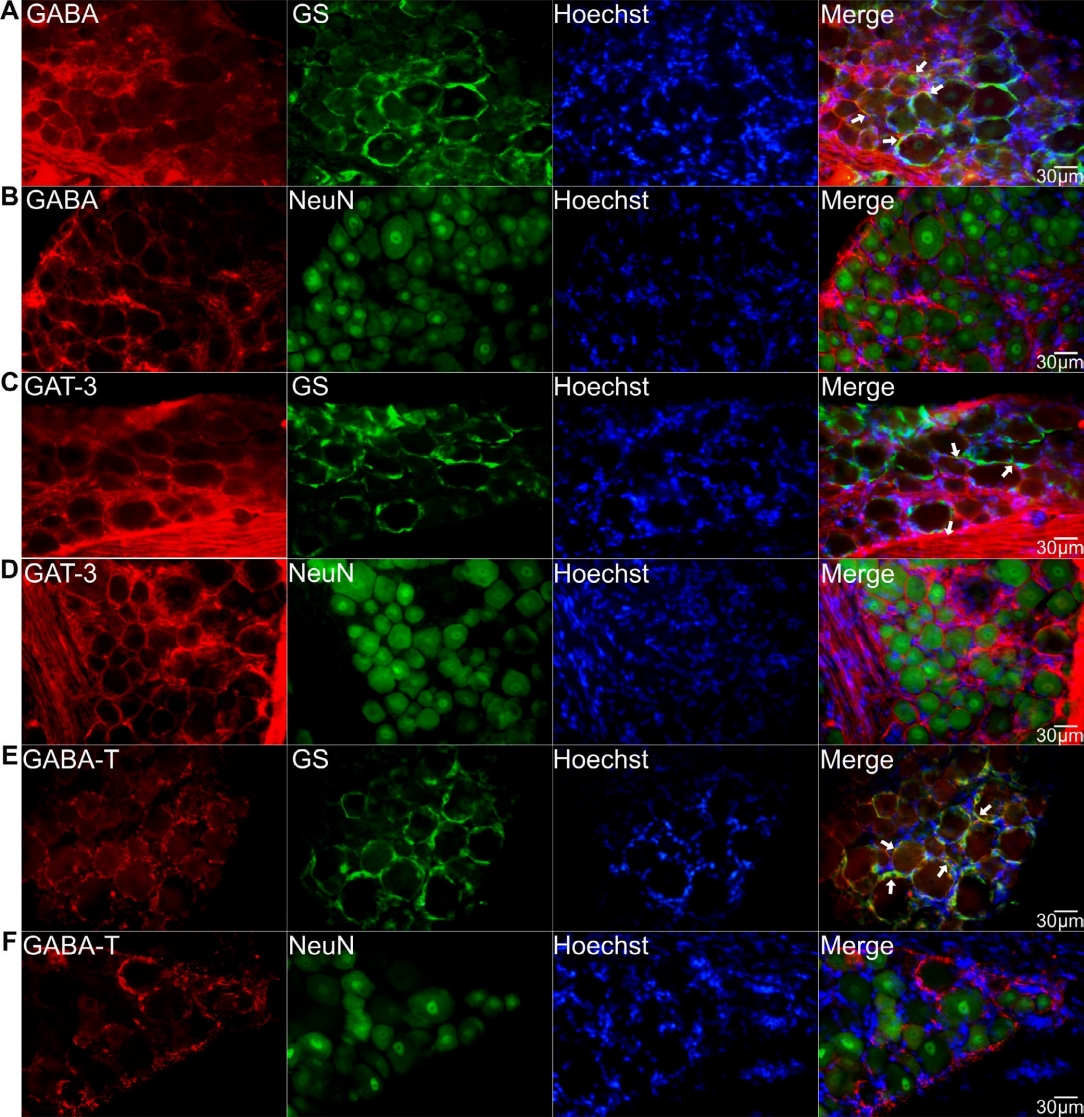
Expression of GABA, GAT‐3, and GABA‐T in the DRG. Panels (A, B) illustrate the staining of GABA in the DRG and its co‐localization with GS in satellite glial cells (SGC), while indicating a lack of expression in NeuN‐positive neurons. Panels (C, D) show the expression of GABA transporter 3 (GAT‐3) in SGC, with no expression detected in neurons stained for NeuN. Finally, panels (E, F) demonstrate the co‐localization of GABA transaminase (GABA‐T) with SGC, but not with NeuN‐positive neuronal elements.

To evaluate the expression of all components involved in GABAergic signaling, we performed immunofluorescence assays on DRG to detect the presence of the GABA transporter (GAT‐3), GABA‐T, and GABA itself. Co‐expression of GABA (Figure 3A), GAT‐3 (Figure 3C), and GABA‐T (Figure 3E) was observed in SGC positive for glutamine synthetase (GS). In contrast, these elements are not expressed in neurons, as evidenced by the lack of co‐localization with the neuronal marker NeuN (Figure 3B,D,F).

**FIGURE 4:**
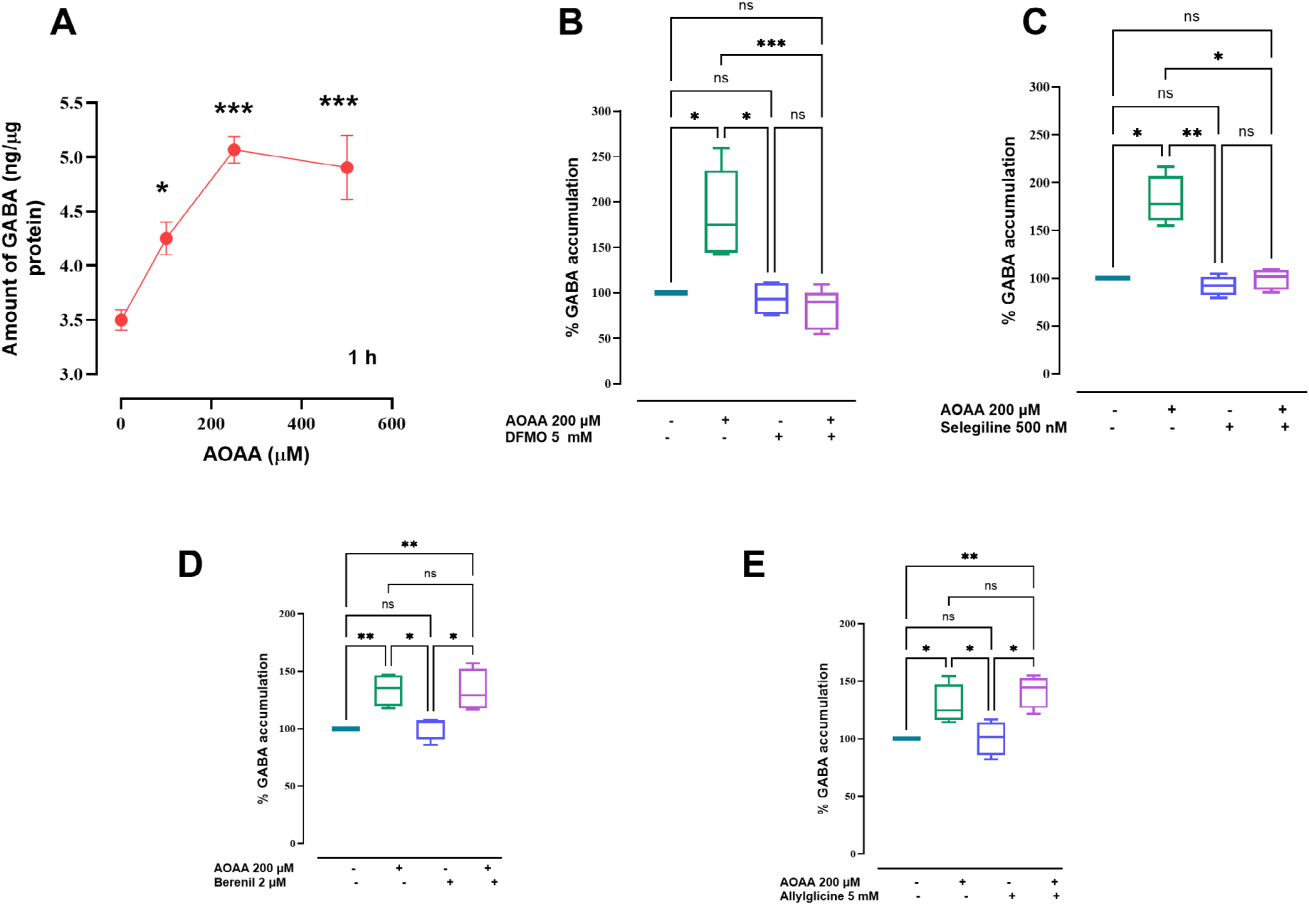
ODC and MAOB, but not DAO or GAD, participate in GABA synthesis in the SGC. Panel (A) shows a typical experiment measuring GABA accumulation induced by GABA‐T inhibition over 1 h, using amino‐oxyacetic acid (AAOA) at various concentrations in SGC cultures. (B) Shows the effect of ODC inhibition with DMFO (5 mM) on AAOA‐induced GABA accumulation at 200 μM during the 1 h. (C) Shows the MAOB inhibition with selegiline (500 nM) on AAOA‐induced GABA accumulation. Selegiline prevents AAOA‐induced accumulation. Parts (D, E) showed the effect of blocking DAO with Berenil (2 μM) and GAD with Allylglycine (5 mM), neither of which modifies AAOA‐induced GABA accumulation. Statistical significance is indicated as *p < 0.05, **p < 0.01, ***p < 0.001, and ns for no significant differences between groups. Data analysis was conducted using the Kruskal‐Wallis test followed by Dunn's test.

To understand the role of the enzymes involved in GABA synthesis, we examined how inhibiting ODC, monoamine oxidase (MAOB), diamino oxidase (DAO), and glutamic acid decarboxylase (GAD) affects endogenous GABA accumulation. This was done by blocking GABA‐T with aminooxy acetic acid (AOAA; Aceves et al. 1992; Löscher et al. 1989) in cultured SGC, under the assumption that all pathways for GABA synthesis and metabolism via GABA‐T are functional. Figure 4A shows GABA accumulation in SGC cultures after a 1‐h pretreatment with various concentrations of AOAA. Maximal accumulation occurred at 300 μM; thus, 200 μM was chosen for subsequent experiments to evaluate the effects of enzyme inhibitors on GABA levels. Figure 4B illustrates how AOAA‐induced GABA accumulation was significantly decreased by inhibition of ODC with DFMO 5 mM (AAOA median 175% rank 142–259 vs. AAOA +DFMO 90% rank 54–109; *p* = 0.002, *n* = 5. Kruskall‐Wallis test), MAOB with selegiline 500 nM (Heinonen and Lammintausta 1991) (AOAA: median 177% rank 154–216 vs. AOAA + Selegiline: 102% rank 85–109; *p* = 0.036, *n* = 4, Kruskall‐Wallis test). DMFO or Selegiline alone did not significantly alter GABA levels compared to control (Control: 100% vs. DMFO median 93% rank 75–111%; *p* = 0.706, *n* = 4; control 100% vs. selegiline median 92% rank 79–104; *p* = 0.369, *n* = 4, Kruskal‐Wallis test).

In contrast, the inhibition of DAO with Berenil 2 μM (Balana‐Fouce et al. 1986) or GAD with allylglycine 5 mM (Horton and Meldrum 1973) had no significant effect on AOAA‐induced GABA accumulation. As shown in Figure 4C,D, GABA levels remained comparable: AOAA: 135% rank 117–146 vs. AOAA + Berenil: 129% rank 116–156 (*p* = 0.881), and AOAA: 124% rank 114–154 vs. AOAA + Allylglycine: 144% rank 121–155 (*p* = 0.6, *n* = 4, Kruskal‐Wallis test).

### 3.2 Best1 Channel Mediates GABA Release in the DRG

It is noteworthy that the activation of the calcium‐activated anion channel Best1 has been previously associated with the release of GABA from glial cells in the brain of rodents and the DRG of turtles. Then, we evaluated the expression of transcripts for Best1 and the vesicular GABA transporter (vGAT), a protein necessary for vesicular loading of the neurotransmitter, to investigate the possibility that GABA release in the DRG may occur via Best1 or vesicular pathways (Joo et al. 2024; Vargas‐Parada et al. 2021; Woo et al. 2018; Yoon et al. 2014).

**FIGURE 5:**
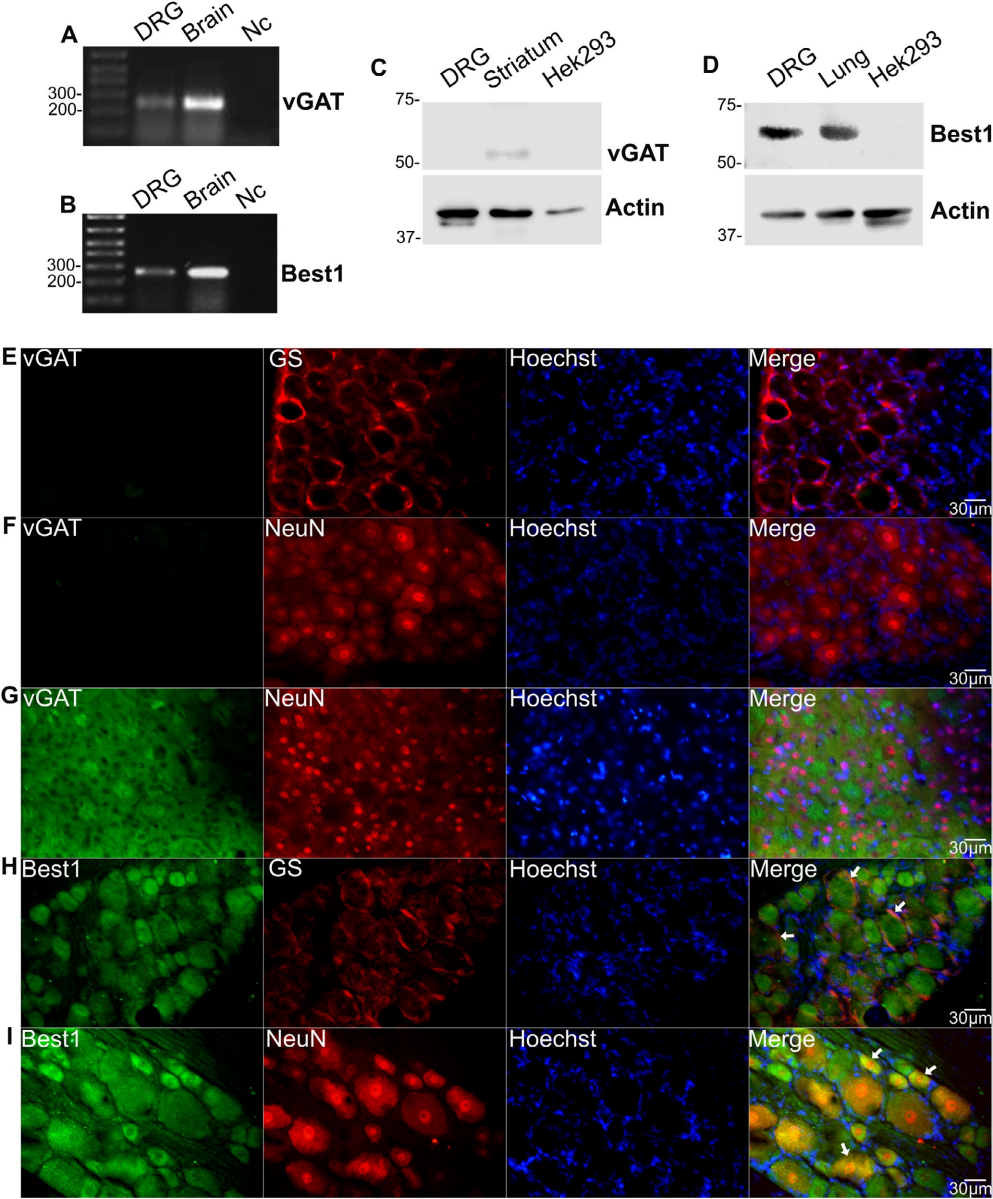
Expression of the vesicular GABA transporter (vGAT) and Bestrophin channel 1 (Best1) in the DRG. Panels (A, B) display a representative blot from the PCR analysis of vGAT and Best1 transcripts obtained from whole DRG mRNA, demonstrating that both transcripts are expressed in the DRG, with whole‐brain mRNA used as a control. Molecular weight markers are on the left. Panel (C) shows the Western blot for vGAT in DRG homogenates, where no positive signal was detected, with striatum as a positive control and HEK293 cells as a negative control. Panel (D) shows a Western blot for Best1 in DRG homogenates, with lung as a positive control and HEK293 cells as a negative control. (E, F) depict the absence of immunohistochemical signals for vGAT in glutamate synthase (GS)‐positive cells and in NeuN‐positive neurons. Panel (G) depicts vGAT‐positive staining in striatal tissue. Finally, Panels H and I illustrate the co‐localization of Best1 in GS‐positive cells and NeuN‐positive elements.

Figure 5A,B show transcript expression levels of vGAT and Best1, respectively, while Figure 5C,D depict Best1 protein expression in DRG homogenates. Protein extracts from the striatum and lung served as positive controls, and extracts from HEK‐293 cells served as negative controls. vGAT protein immunostaining was not present in GS‐positive glial cells and NeuN‐positive neurons, according to immunofluorescence analysis (Figure 5E–G). Remarkably, as shown in Figure 5H,I, Best1 was found in both neurons and satellite glial cells (SGC).

**FIGURE 6:**
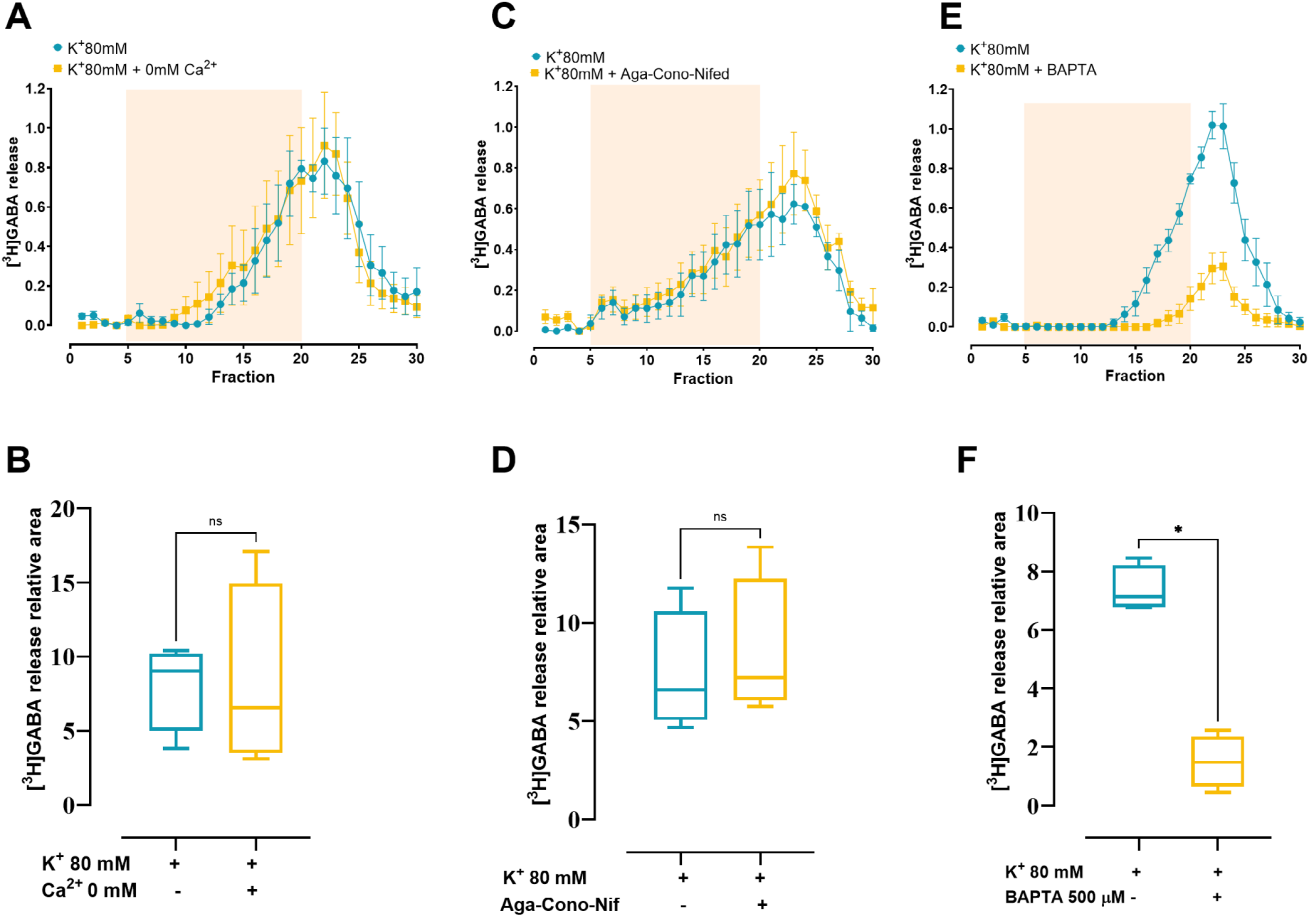
K^+^‐induced [^3^H]GABA release in the DRG depends on intracellular Ca^2+^. The levels of [^3^H]GABA released are expressed as a fraction of GABA above the basal. The area under the curve (UCA) from fractions 5 to 30 is presented in the bar plot below the curves. Panel (A, B) Shows GABA release during K^+^‐induced GABA release under control conditions and in the absence of external Ca^2+^ in a perfusion solution that has been equimolarly substituted with Mg^2+^. Panel (C, D) Illustrates the effects of Ca^2+^ channel blockers, including Agatoxin‐TK (Aga), *ω*‐conotoxin GVIA (Cono) pretreatment, and nifedipine (Nif), in the perfusion solution on K^+^‐induced GABA release. Panel (E, F) Shows the effect of preincubating the DRG with the Ca^2+^ chelator BAPTA (500 μM) on K^+^‐induced GABA release. *p < 0.05, ns indicates no significant difference, determined by the Mann–Whitney test.

Although the release of GABA from neurons is mediated by high‐voltage activated Ca^2+^ (HVA) channels and is reliant on extracellular Ca^2+^ (Catterall 1999), glial GABA release, as previously mentioned, can occur via reversal transporters (Lee et al. 2010; Yoon et al. 2014) or Best1 channels (Oh and Lee 2017; Orrego 1980). Therefore, we next measured K^+^‐induced [^3^H]GABA release in order to describe the mechanism of GABA release in the DRG. A depolarizing pulse of 80 mM K^+^ (fractions 5–20) progressively promoted GABA release, peaking at fraction 23 before settling back to baseline, as shown in Figure 6A. Replacing extracellular Ca^2+^ with equimolar Mg^2+^ did not significantly affect release dynamics or total GABA release (80 mM K^+^ median: 9.05 rank 3.84–10.40 vs. 80 mM K^+^ + 0 Ca^2+^: 6.57 rank 3.11–17.08; *p* = 0.685, *n* = 4, Mann–Whitney test; Figure 6B). Furthermore, blocking HVA channels with *ω*‐Agatoxin IVA, *ω*‐Conotoxin GVIA, and nifedipine did not significantly alter GABA release (80 mM K^+^: 6.59 rank 4.68–11.76 vs. 80 mM K^+^ + toxins: 7.21 rank 5.74–13.84; *p* = 0.57, *n* = 4, Mann–Whitney test; Figure 6C,D), suggesting that classical neuronal release pathways do not mediate GABA release in the DRG.

**FIGURE 7:**
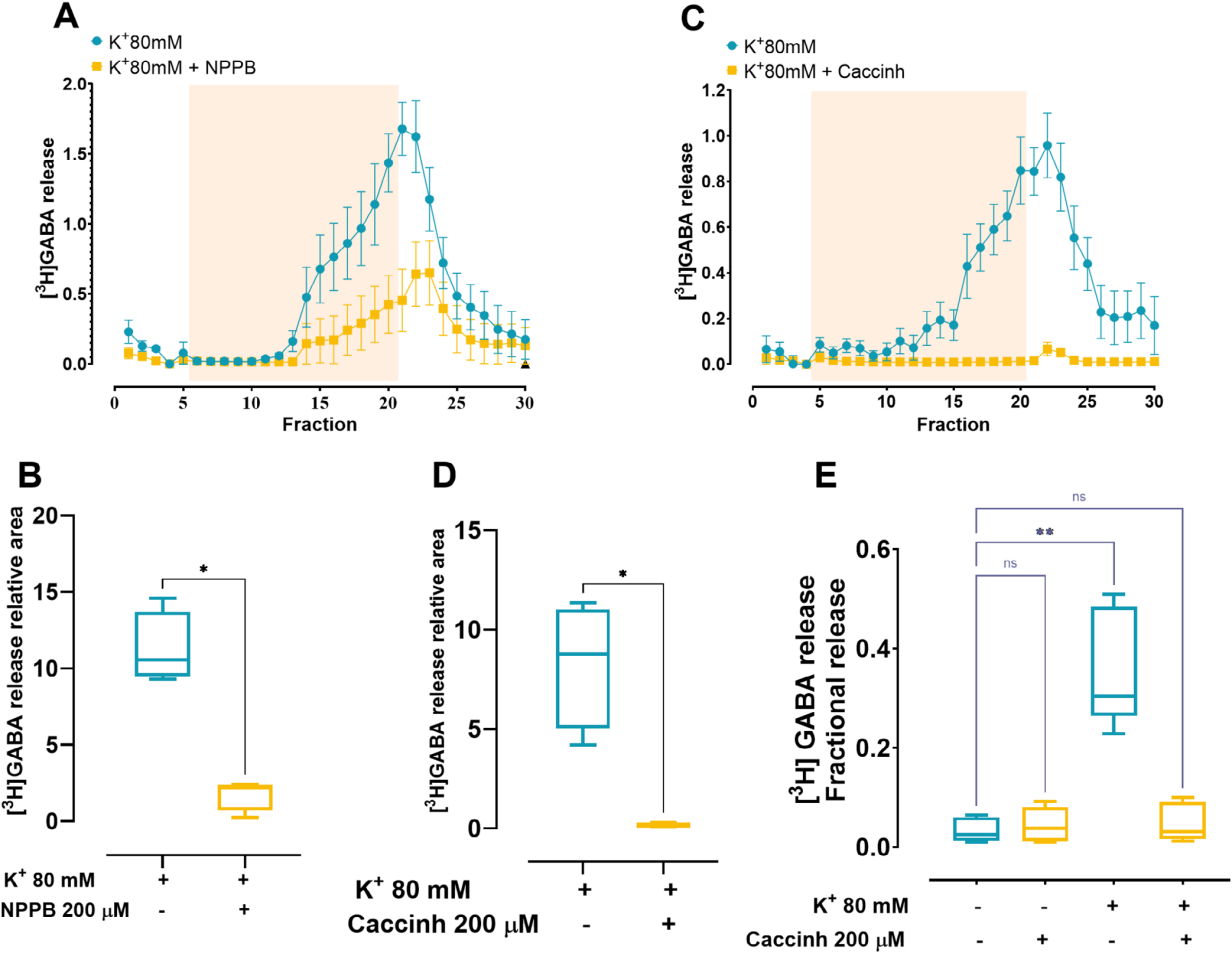
K^+^‐induced [^3^H]GABA release in the DRG and SGC may occur through Best1 channels. Panels (A, C) show the effects of the channel blockers NPPB and CaCCinh on GABA release, respectively. The area under the curve (AUC) from fractions 5 to 30 is presented in the bar plot below the curves. Panel (B, D) *p < 0.05, determined by the Mann–Whitney test. Panel (E) Shows the effect of CaCCinh on GABA release elicited in SGC cultures *p < 0.05, determined by the Kruskal‐Wallis test followed by Dunns' post hoc test.

To examine the function of intracellular Ca^2+^, DRG tissue was preincubated using the Ca^2+^ chelator BAPTA. The results of the analysis (Figure 6E,F) show that BAPTA treatment significantly reduced K^+^‐induced GABA release by ~75% (80 mM K^+^: 7.13 rank 6.76–8.46 vs. 80 mM K^+^ + BAPTA: 1.48 rank 0.44–2.56; *p* = 0.028, *n* = 4, Mann–Whitney test). Next, we evaluated the role of Best1 in GABA release using two pharmacological blockers, NPPB and CaCCinh. Both agents significantly reduced GABA release, with CaCCinh producing a near‐complete inhibition (Figure 7). Specifically, NPPB reduced GABA release from 10.55 rank 9.26–14.57 to 2.19 rank 0.23–2.41 (*p* = 0.014, *n* = 4; Figure 7B). Consistent with this, CaCCinh decreased release from 8.67 rank 4.19–11.63 to 0.22 rank 0.01–0.29 (*p* = 0.028, *n* = 4), supporting the idea of a significant contribution of Best1 channels in non‐vesicular GABA release in the DRG.

To evaluate whether GABA release from SGC is modulated by the BEST1 channel, we performed experiments in SGC cultures. In Figure 7C, CaCCinh decreases high K^+^ (80 mM)‐stimulated [^3^H]GABA release to values near the control (control 0.024 rank 0.01 to 0.064 vs. high K^+^ 0.30 rank 0.23 to 0.51) (*p* = 0.003, *n* = 5) and vs. high K^+^+CaCCinh 0.031 rank 0.012 to 0.1 (*p* = 0.57, *n* = 5).

### 3.3 Modulation of DRG Neuron Excitability by Extrasynaptic GABA_A_ Receptors Depends on GABA Synthesized and Released From Satellite Glial Cells

Our findings suggest that GABA is synthesized in the DRG via the ornithine–putrescine pathway and is released through the Best1 channels. We next investigated the impact of the described experimental maneuvers on the amplitude of cAP evoked in sensory neurons in order to ascertain whether inhibition of important enzymes involved in GABA synthesis, or the Best1‐mediated blockade of GABA release, influences neuronal excitability. These experiments aimed to evaluate the role of endogenous GABA in tonic modulation of DRG excitability (Hernández‐Reyes et al. 2019).

**FIGURE 8:**
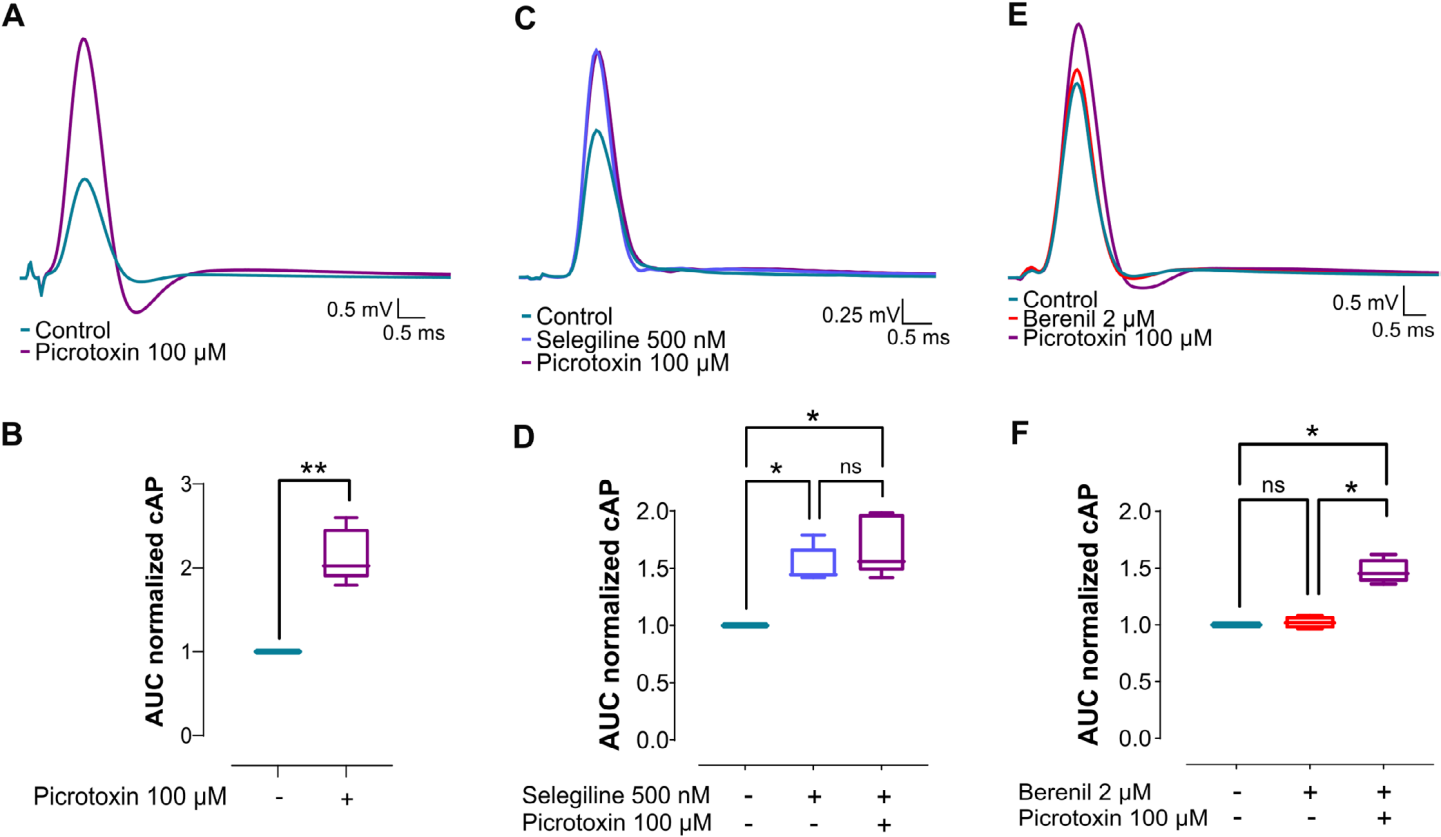
Effects of picrotoxin, selegiline, and berenil on cAP amplitude in the DRG neurons. Panel (A) shows superimposed voltage traces (cAP) in the presence and absence of picrotoxin (100 μM). In all cases, the comparison of the area under the curve (AUC) is presented in the bar plot below the traces. Panels (C, D) show that selegiline (500 nM), which blocks MAOB, increases the cAP, and picrotoxin occludes its effect. Panels (E, F) indicate that the inhibition of DAO with berenil does not modify cAP amplitude. In contrast, the addition of picrotoxin significantly increases cAP amplitude and, consequently, the area under the curve. Statistical significance is denoted as *p < 0.05, **p < 0.01, and ns indicates no significant differences, with the Wilcoxon test used in panel (B) and the Friedman test followed by Dunn's test applied to the data in panels (D, F).

GABAergic shunting inhibition was evident when picrotoxin (100 μM) was used to block GABA_A_ receptors, as shown in Figure 8A,B. The cAP amplitude increased significantly from 1.00 to 2.02, with a range of 1.79–2.6, in the control and picrotoxin conditions, respectively (*p* = 0.0312, *n* = 5; Wilcoxon test). Similarly, inhibition of MAOB with selegiline (500 nM) produced a significant increase in cAP amplitude (Figure 8C,D; control: 1.00 vs. selegiline: 1.44 with a range 1.41–1.79; *p* = 0.027, *n* = 5; Friedman test following Dunn's). Importantly, subsequent application of picrotoxin did not produce a further significant increase in cAP amplitude (selegiline: 1.44 rank 141–179 vs. selegiline + picrotoxin: 1.56 rank 1.42–1.98; *p* = 0.75, *n* = 5; Friedman test following Dunn's), suggesting that both drugs share the same GABAergic inhibitory mechanism or that a saturation effect may limit further modulation.

In contrast, inhibiting enzymes not involved in GABA synthesis in the DRG had no effect. Blocking DAO with berenil (2 μM) did not significantly change the cAP amplitude compared to control (Figure 8E,F; control: 1.00 vs. berenil: 1.08 rank 0.96–1.08; *p* = 0.034, *n* = 4). However, subsequent application of picrotoxin significantly increased cAP amplitude (berenil: 1.02 rank 0.96–1.08 vs. berenil + picrotoxin: 1.45 rank 1.36–1.62; *p* = 0.034, *n* = 4).

**FIGURE 9:**
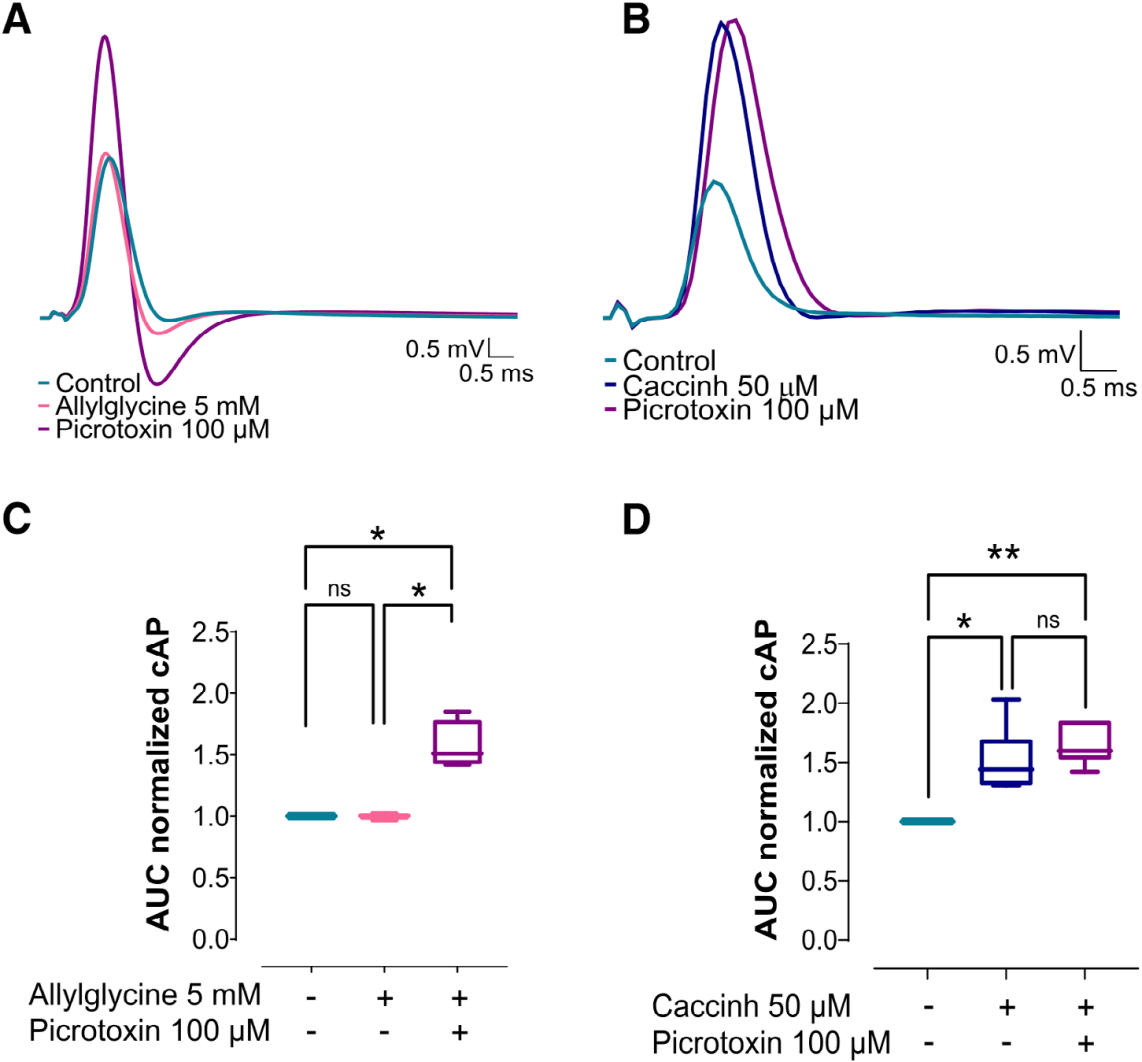
Effects of allylglycine and CaCCinh on cAP amplitude in the DRG neurons. Panel (A, B) shows that blocking GAD65/67 with allylglycine did not alter cAP amplitude. In all cases, the comparison of the area under the curve (AUC) is presented in the bar plot below the traces. Panel (C, D), incubation with the Best1 channel blocker CaCCinh increases cAP amplitude. In both cases, the application of allylglycine and CaCCinh was followed by the application of Picrotoxin, as indicated. Statistical significance is denoted as *p < 0.05, **p < 0.01, and ns indicates no significant differences after the Friedman test followed by Dunn's test.

On the other hand, inhibition of GAD with allylglycine (5 mM) did not affect cAP amplitude (Figure 9A,B; control: 1.00 vs. allylglycine: 1.02 rank 0.97–1.02; *p* > 0.99, *n* = 4), but addition of picrotoxin significantly increased it (allylglycine: 1.02 rank 0.97–1.02 vs. allylglycine + picrotoxin: 1.51 rank 1.42–1.85; *p* = 0.033, *n* = 4), reinforcing the presence of a tonic GABAergic inhibition originated from non‐glutamic acid pathways. Finally, blockade of the Best1 channel with CaCCinh (50 μM) significantly increased cAP amplitude (Figure 9C,D; control: 1.00 vs. CaCCinh: 1.44 rank 1.30–2.03; *p* = 0.001, *n* = 6). The addition of picrotoxin did not significantly enhance this effect (CaCCinh: 1.44 rank 1.30–2.03 vs. CaCCinh + picrotoxin: 1.6 rank 1.42–2.35; *p* = 0.248, *n* = 6). Overall, these findings support a model in which GABA synthesized through the ornithine–putrescine pathway in SGC is released via the Best1 channel and acts on extrasynaptic GABA_A_ receptors to tonically modulate neuronal excitability in the DRG.

**FIGURE 10:**
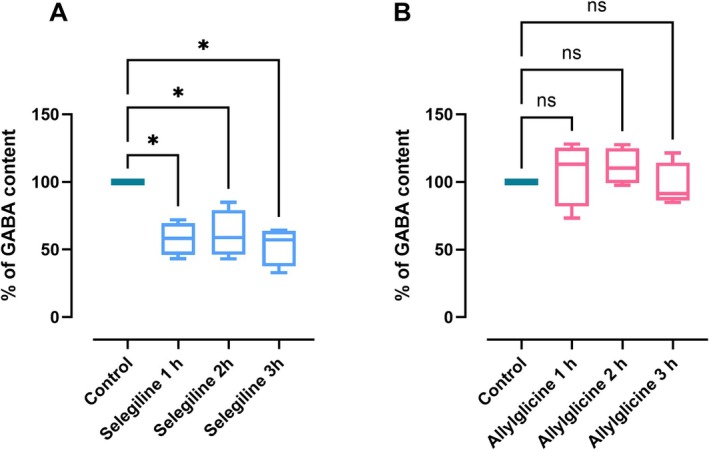
Treatment with selegiline, but not allylglycine, decreases GABA content in the DRG. (A) Shows that the MAOB inhibitor selegiline (500 nM) decreases GABA content in the DRG for up to 3 h, whereas in (B), the GAD inhibitor allylglycine (5 mM) did not modify GABA content. *p < 0.05, and ns indicates no significant difference analyzed using the Kruskal‐Wallis test followed by Dunn's test.

Finally, to test whether the blockade of MAOB or GAD modifies whole DRG GABA content, we incubate ganglia with selegiline or allylglycine and determine GABA by HPLC at 1, 2, or 3 h of treatment. DRG incubation with selegiline decreases GABA content to 50% approximately in 1 h of treatment, and it remains up to 3 h (Figure 10A) control 100 vs. selegiline 3 h 57.13 rank 32–64, *p* = 0.028, *n* = 4, Friedman's ANOVA (post hoc analysis: Dunn's test); in contrast, allylglycine did not modify content within the 3 h period (Figure 10B control 100 vs. allylglycine 3 h 91.52 rank 84–121, *p* > 0.999, *n* = 4, Friedman test following by Dunn's).

## 4 Discussion

Our data indicate that in the DRG, GABA is synthesized by satellite glial cells (SGC) via the ornithine–putrescine pathway and is subsequently released through the Bestrophin‐1 (Best1) channel. The released GABA activates extrasynaptic GABA_A_ receptors on sensory neurons, modulating their excitability. Our results also agree with the idea that the DRG takes part in a loop that includes GABA synthesis, release, receptor activation, neuronal activity modulation, uptake, and degradation. This regulatory loop seems to regulate neuronal excitability and, in turn, the transmission of sensory data to the spinal cord in a homeostatic manner.

### 4.1 Synthesis of GABA in SGC of the DRG Regulates Neuronal Excitability

Because of its action on extrasynaptic GABA_A_ *α*_5_ receptors, GABA is crucial for regulating the excitability of sensory neurons (Feltz and Rasminsky 1974; Loeza‐Alcocer et al. 2013). It is debatable, though, where this GABA came from. Based on the presence of transcripts and protein for the vesicular GABA transporter (vGAT), mRNA for glutamic acid decarboxylase (GAD), the enzyme that converts glutamate to GABA, and GABA‐containing vesicles within neurons in the DRG, some results support a neuronal origin (Du et al. 2017). However, there is evidence that SGC are the main source of GABA in the DRG due to the expression of GABA as well as Best1 channels and their capacity to control GABA release (Minchin and Iversen 1974; Vargas‐Parada et al. 2021).

Our results corroborate that the ornithine‐putrescine pathway, a well‐established metabolic pathway in the glial cell lineage, is the mechanism by which SGC synthesize GABA in the DRG (Jo et al. 2014; Yoon et al. 2014). Only SGC, not neurons, are able to uptake GABA in the DRG, according to autoradiographic research using [^3^H]GABA since the 1970s (Schon and Kelly 1974a). Our immunofluorescence evidence does not support neuronal GABA synthesis, but it is still possible that this GABA comes from neurons and is then taken up by SGC through GABA transporters (Schon and Kelly 1974b). In particular, DRG neurons did not exhibit immunoreactivity to anti‐GABA antibodies, in contrast to neurons that are known to generate GABA via canonical pathways (Figure 2C; Dacks et al. 2013; Yu et al. 2021).

In further support of a glial origin, transcripts encoding enzymes of the ornithine–putrescine pathway (ODC, MAOB, and DAO) were all detected in the DRG (Figure 1). Protein expression analyses revealed that ODC and DAO were present in neurons and SGC, whereas MAOB was expressed exclusively in SGC. Importantly, GABA accumulation in SGC cultures was suppressed by pharmacologically inhibiting ODC with DMFO and MAOB with selegiline, while DAO (with Berenil) or GAD (with allylglycine) showed no effect (Balana‐Fouce et al. 1986; Fowler et al. 1980; Horton and Meldrum 1973; Yoon et al. 2014). Although MAOB inhibition causes a decrease in GABA levels, we know this is not direct evidence. However, we consider that GABA is synthesized by this enzyme because there is considerable evidence in the literature that MAOB synthesizes GABA in glial cells (Cho et al. 2021; Chun et al. 2022; Ju et al. 2024; Lee et al. 2025; Yoon et al. 2014), and conversely, there is no evidence that MAOB inhibition modifies cellular metabolism by altering GABA levels through any other mechanism. This implies that SGC produce GABA through the ornithine–putrescine pathway's ODC and MAOB‐dependent metabolism, a process that has been shown to occur in astrocytes before (Kwak et al. 2020; Yoon et al. 2014).

Though DAO and ODC are present in DRG neurons, their known role in other metabolic pathways (McGrath et al. 2009; Pegg 2006; Raboni et al. 2010), and the lack of effect of DAO inhibition on the cAP (Figure 6) argue against a neuronal contribution to GABA synthesis. Furthermore, despite the presence of mRNA for GAD65 and GAD67, neither protein was detected by Western blot or immunofluorescence in our hands (Figure 2), casting doubt on the notion of neuronal GABA synthesis. Previous studies have validated the antibodies used in these experiments in brain tissue and confirmed their specificity (Figure 3; Dacks et al. 2013; Yu et al. 2021). This discrepancy may be attributed to post‐transcriptional repression mechanisms, such as microRNA‐mediated silencing, which has been shown to decrease GAD expression in other systems, and to the absence of vGAT expression. However, the lack of protein, but not mRNA, can be explained by microRNA silencing (Kołosowska et al. 2023; Leitão and Enguita 2022; Ma et al. 2016; Manrique et al. 2009; Pillai et al. 2007).

When taken as a whole, these findings provide compelling evidence that the SGC are the main source of GABA in the DRG, which controls neuronal excitability by activating extrasynaptic GABA_A_ receptors (Figure 5).

### 4.2 Mechanism of GABA Release in the DRG

We measured [^3^H]GABA release in response to depolarization with high extracellular K^+^ in order to look into the origin and mechanism of GABA release in the DRG. K^+^‐induced depolarization is still a commonly used technique to study neurotransmitter release mechanisms, despite the fact that it cannot accurately mimic physiological conditions (Minchin 1975; Minchin and Iversen 1974). However, it is also worth noting that the results of this experimental approach frequently agree with electrophysiological data, evidencing its practicality in experiments.

Previously, it has been shown that the cellular composition of the sample affects the threshold and dynamics of K^+^‐induced GABA release. Lower K^+^ concentrations are sufficient to induce neurotransmitter release in purely neuronal preparations. This release is usually vesicular and dependent on extracellular Ca^2+^ (Neal and Bowery 1979; Orrego 1980). However, GABA release is typically Ca^2+^‐independent and necessitates higher K^+^ concentrations in glial‐enriched or mixed preparations, indicating non‐vesicular mechanisms (Minchin and Iversen 1974; Sellström and Hamberger 1976; Vargas et al. 1977).

A gradual increase in [^3^H]GABA release induced by high K^+^ (80 mM) was observed in the DRG preparations used in our studies, peaking at approximately 15 min (Figure 5C). These data contrast with the faster‐release kinetics previously reported for glial cell cultures, most likely reflecting the complex cell composition of the DRG tissue, which includes neurons, SGC, and connective tissue (Albrecht et al. 1998). Interestingly, experiments performed in the turtle DRG have shown a similar time course (Vargas‐Parada et al. 2021). To determine whether the GABA released originated from SGC or neurons, we used two different approaches. The first consisted of the removal of extracellular Ca^2+^, and the second was the pharmacological blockade of high‐voltage‐activated Ca^2+^ channels of the N‐, P/Q‐, and L‐type, using *ω*‐conotoxin GVIA, *ω*‐agatoxin IVA, and nifedipine, respectively. K^+^‐evoked GABA release was not significantly changed by either manipulation, indicating that the release was non‐vesicular and extracellular Ca^2+^‐independent (Figure 6). These results refute a neuronal source and support earlier findings that glial cells are the primary source of high K^+^‐evoked GABA release in comparable situations (Orrego 1980; Sellström and Hamberger 1976). Although the precise mechanism of glial GABA release during high‐K^+^ depolarization remains unclear (Bowery et al. 1979), elevated intracellular Ca^2+^ is thought to drive it (Verkhratsky and Kettenmann 1996).

Despite the existence of GABA‐containing vesicles in DRG neurons (Du et al. 2017), our release assays indicate that these vesicles are not the primary source of functional GABA release in depolarizing circumstances. Instead, our data support a model where SGC are the primary source of tonic GABA release that regulates the excitability of DRG neurons through the Best1 channel, and that this release depends on intracellular Ca^2+^. The observation that isolated SGC in culture exhibit K^+^‐induced [^3^H]GABA release, and that this release is sensitive to Best1 channel blockade, strongly supports this conclusion.

### 4.3 Functional Implications

Previous studies from our group and others have demonstrated that the extrasynaptic GABA_A_ *α*_5_ receptor is expressed in sensory neurons (Bravo‐Hernández et al. 2016; Loeza‐Alcocer et al. 2013). Activation of these extrasynaptic GABA_A_ receptors tonically modulates sensory neuron excitability through two opposing mechanisms: one that increases excitability and another that decreases it. The excitatory effect results from tonic depolarization driven by sustained chloride (Cl^−^) efflux, as the Cl^−^ equilibrium potential is depolarized due to the activity of the NKCC1 cotransporter (Price et al. 2005). Conversely, the inhibitory effect is attributed to a shunting mechanism (often called a “neuronal short circuit”) caused by increased Cl^−^ conductance (Mitchell and Silver 2003).

Our findings indicate that the GABA responsible for the tonic activation of these extrasynaptic receptors is present within the DRG. This was evidenced by the application of 100 μM picrotoxin, a concentration known to block the activity of all GABA_A_ receptor subtypes (Farrant and Nusser 2005). The increased cAP amplitude that follows this blockade, which reflects changes in cell excitability, supports the notion that a tonic Cl^−^ conductance through GABA_A_ receptors exerts an inhibitory effect on sensory neurons under physiological conditions (Castro et al. 2011; Hernández‐Reyes et al. 2019). The differences in the shape of the cAP waveform arise because the differential amplifier outputs the difference between positive‐ and negative‐phase signals.

We next inhibited the enzymes that synthesize GABA and evaluated their effect on the cAP in order to investigate whether GABA derived from SGC functionally activates the extrasynaptic receptors. Notably, selegiline‐induced MAOB inhibition increased cAP amplitude, a result not observed when diamine DAO or GAD inhibition was used. As mentioned earlier, based on our immunofluorescence and GABA accumulation data showing a selective expression of MAOB in our cultured SGC, we propose that SGC are the primary source of ambient GABA that tonically controls the excitability of the sensory neurons. The findings of HPLC assays showing a drop in ambient GABA following MAOB inhibition but not GAD inhibition (Figure 10) are also in line with this interpretation, as well as previous reports (Yoon et al. 2014; Ju et al. 2024), here. Furthermore, GABA appears to be released through Best1 channels, as blockade of these channels also facilitated the cAP amplitude, a conclusion that is consistent with data reported in previous studies (Lee et al. 2010; Vargas‐Parada et al. 2021).

In summary, the facilitation of the cAP observed after inhibition of MAOB and blockade of the Best1 channel was not further enhanced by the subsequent application of picrotoxin, unlike the facilitation observed when picrotoxin was administered alone (Figure 7A,B). In contrast, the inhibition of DAO (Figure 7E,F) and GAD (Figure 7G,H) did not increase cAP facilitation. However, such an effect was observed after subsequent picrotoxin administration.

Inhibition of the MAOB enzyme with selegiline resulted in an increased cAP evoked by electrical stimulation of A*β* fibers at a site near the soma in the DRG. Considering that Schwann cells, which express GAD67 and synthesize GABA, are present along all non‐nociceptive afferent fibers, we propose that the GABA responsible for shunting these fibers via extrasynaptic GABA_A_ receptors originates from SGC. This proposal is consistent with recent findings showing that GABA‐activating GABA_A_ *α*_5_ receptors are of astrocytic origin and synthesized via MAOB. According to these authors, MAOB inhibition decreased ambient GABA levels, which in turn decreased the tonic current mediated by these receptors and, most notably, produced analgesia in a neuropathic pain model (Ju et al. 2024). Additionally, these data support our previous work, which showed that GABA_A_ *α*_5_ receptors are pronociceptive in chronic pain, as their blockade produces analgesia across various pain models (Bravo‐Hernández et al. 2016; Hernández‐Reyes et al. 2019; De la Luz‐Cuellar et al. 2019). Therefore, we conclude that SGC synthesize GABA via MAOB, which is then released through Best1 channels, contributing to the regulation of sensory neuron excitability.

## Author Contributions

Conceptualization: N.J.‐B., R.D.‐L., B.F.G., R.F. and J.S.V.; Data curation: N.J.‐B., R.G.‐R., F.J.P.‐B., G.R., R.D.‐L., and B.F.G.; Formal analysis: N.J.‐B., R.D.‐L., J.S.V. and R.F.; Investigation: N.J.‐B., R.D.‐L., and B.F.G.; Methodology: N.J.‐B., R.D.‐L., B.F.G., and R.G.‐R.; Validation: N.J.‐B., R.D.‐L., B.F.G., R.F., and J.S.V.; Writing: N.J.‐B., R.D.‐L., B.F.G., and R.F.; Review: N.J.‐B., R.D.‐L.; B.F.G., R.F., and J.S.V.; Editing: N.J.‐B., R.D.‐L., and B.F.G.

## Funding

This research was partially funded by “Secretaria de Educación, Ciencia y Tecnología de la Ciudad de México, grant # SECTEI/146/2024” awarded to RF.

## Ethics Statement

The Institutional Animal Care Committee of Cinvestav approved the animal study. Approval Code: 0146‐15 Approval Date: 9/1/2021 to 12/31/2024.

## Conflicts of Interest

The authors declare no conflicts of interest.

## Supporting information


**Table S1:** Sequences of oligonucleotides RT‐PCR.

**Table S2:** Antibodies for immunofluorescence and western blot.

## Data Availability

The data that support the findings of this study are available from the corresponding author upon reasonable request.

## References

[glia70190-bib-0001] Aceves, J. , B. Floran , D. Martinez‐Fong , J. Benitez , A. Sierra , and G. Flores . 1992. “Activation of D1 Receptors Stimulates Accumulation of *γ*‐Aminobutyric Acid in Slices of the Pars Reticulata of 6‐Hydroxydopamine‐Lesioned Rats.” Neuroscience Letters 145, no. 1: 40–42. 1461565 10.1016/0304-3940(92)90198-g

[glia70190-bib-0002] Albrecht, J. , A. Bender , and M. Norenberg . 1998. “Potassium‐Stimulated GABA Release Is a Chloride‐Dependent but Sodium‐and Calcium‐Independent Process in Cultured Astrocytes.” Acta Neurobiologiae Experimentalis 58, no. 3: 169–175. 9803010 10.55782/ane-1998-1271

[glia70190-bib-0003] Balana‐Fouce, R. , T. G. Pulido , D. Ordonez‐Escudero , and A. Garrido‐Pertierra . 1986. “Inhibition of Diamine Oxidase and S‐Adenosylmethionine Decarboxylase by Diminacene Aceturate (Berenil).” Biochemical Pharmacology 35, no. 9: 1597–1600. 3085681 10.1016/0006-2952(86)90130-9

[glia70190-bib-0004] Beart, P. , J. Kelly , and F. Schon . 1974. γ‐Aminobutyric Acid in the Rat Peripheral Nervous System, Pineal and Posterior Pituitary. Portland Press Ltd.

[glia70190-bib-0005] Bowery, N. G. , D. A. Brown , R. D. White , and G. Yamini . 1979. “[3H]Gamma‐Aminobutyric Acid Uptake Into Neuroglial Cells of Rat Superior Cervical Sympathetic Ganglia.” Journal of Physiology 293: 51–74. 501628 10.1113/jphysiol.1979.sp012878 PMC1280702

[glia70190-bib-0006] Bravo‐Hernández, M. , J. A. Corleto , P. Barragán‐Iglesias , et al. 2016. “The *α*5 Subunit Containing GABA_A_ Receptors Contribute to Chronic Pain.” Pain 157, no. 3: 613–626. 26545088 10.1097/j.pain.0000000000000410 PMC4950669

[glia70190-bib-0007] Bustamante, H. A. , M. F. Ehrich , and B. G. Klein . 2021. “Intracellular Potassium Depletion Enhances Apoptosis Induced by Staurosporine in Cultured Trigeminal Satellite Glial Cells.” Somatosensory & Motor Research 38, no. 3: 194–201. 34187291 10.1080/08990220.2021.1941843

[glia70190-bib-0008] Capuano, A. , A. De Corato , L. Lisi , G. Tringali , P. Navarra , and C. D. Russo . 2009. “Proinflammatory‐Activated Trigeminal Satellite Cells Promote Neuronal Sensitization: Relevance for Migraine Pathology.” Molecular Pain 5: 1744–8069. 10.1186/1744-8069-5-43 PMC2731738 19660121

[glia70190-bib-0009] Castro, A. , J. Aguilar , C. Andrés , R. Felix , and R. Delgado‐Lezama . 2011. “GABA_A_ Receptors Mediate Motoneuron Tonic Inhibition in the Turtle Spinal Cord.” Neuroscience 192: 74–80. 21745544 10.1016/j.neuroscience.2011.06.073

[glia70190-bib-0010] Catterall, W. A. 1999. “Interactions of Presynaptic Ca^2+^ Channels and Snare Proteins in Neurotransmitter Release.” Annals of the New York Academy of Sciences 868, no. 1: 144–159. 10414292 10.1111/j.1749-6632.1999.tb11284.x

[glia70190-bib-0012] Cho, H. U. , S. Kim , J. Sim , et al. 2021. “Redefining Differential Roles of MAO‐A in Dopamine Degradation and MAO‐B in Tonic GABA Synthesis.” Experimental & Molecular Medicine 53, no. 7: 1148–1158. 34244591 10.1038/s12276-021-00646-3 PMC8333267

[glia70190-bib-0013] Chun, H. , J. Lim , K. D. Park , and C. J. Lee . 2022. “Inhibition of Monoamine Oxidase B Prevents Reactive Astrogliosis and Scar Formation in Stab Wound Injury Model.” Glia 70, no. 2: 354–367. 34713936 10.1002/glia.24110

[glia70190-bib-0014] Dacks, A. M. , V. Reale , Y. Pi , et al. 2013. “A Characterization of the *Manduca sexta* Serotonin Receptors in the Context of Olfactory Neuromodulation.” PLoS One 8, no. 7: e69422. 23922709 10.1371/journal.pone.0069422 PMC3726668

[glia70190-bib-0015] De la Luz‐Cuellar, Y. E. , E. J. Rodríguez‐Palma , Ú. Franco‐Enzástiga , A. B. Salinas‐Abarca , R. Delgado‐Lezama , and V. Granados‐Soto . 2019. “Blockade of Spinal *α*5‐ GABAA Receptors Differentially Reduces Reserpine‐Induced Fibromyalgia‐Type Pain in Female Rats.” European Journal of Pharmacology 858: 172443. 31181208 10.1016/j.ejphar.2019.172443

[glia70190-bib-0016] Du, X. , H. Hao , Y. Yang , et al. 2017. “Local GABAergic Signaling Within Sensory Ganglia Controls Peripheral Nociceptive Transmission.” Journal of Clinical Investigation 127, no. 5: 1741–1756. 28375159 10.1172/JCI86812 PMC5409786

[glia70190-bib-0017] Farrant, M. , and Z. Nusser . 2005. “Variations on an Inhibitory Theme: Phasic and Tonic Activation of GABA_A_ Receptors.” Nature Reviews Neuroscience 6, no. 3: 215–229. 15738957 10.1038/nrn1625

[glia70190-bib-0018] Feltz, P. , and M. Rasminsky . 1974. “A Model for the Mode of Action of GABA on Primary Afferent Terminals: Depolarizing Effects of GABA Applied Iontophoretically to Neurones of Mammalian Dorsal Root Ganglia.” Neuropharmacology 13, no. 6: 553–563. 4153679 10.1016/0028-3908(74)90145-2

[glia70190-bib-0019] Floran, B. , C. Barajas , L. Floran , D. Erlij , and J. Aceves . 2002. “Adenosine A1 Receptors Control Dopamine D1‐Dependent [^3^H] GABA Release in Slices of Substantia Nigra Pars Reticulata and Motor Behavior in the Rat.” Neuroscience 115, no. 3: 743–751. 12435413 10.1016/s0306-4522(02)00479-7

[glia70190-bib-0020] Fowler, C. J. , L. Oreland , J. Marcusson , and B. Winblad . 1980. “Titration of Human Brain Monoamine Oxidase‐A And‐B by Clorgyline and l‐Deprenil.” Naunyn‐Schmiedeberg's Archives of Pharmacology 311: 263–272. 6771658 10.1007/BF00569406

[glia70190-bib-0021] Gottesfeld, Z. , J. Kelly , and F. Schon . 1973. “Uptake Of‐Aminobutyric Acid (GABA) by Sensory Root Ganglia.” British Journal of Pharmacology 47, no. 3: 640P. PMC1776342 4730854

[glia70190-bib-0022] Hanani, M. 2005. “Satellite Glial Cells in Sensory Ganglia: From Form to Function.” Brain Research. Brain Research Reviews 48, no. 3: 457–476. 15914252 10.1016/j.brainresrev.2004.09.001

[glia70190-bib-0023] Hanani, M. , and A. Verkhratsky . 2021. “Satellite Glial Cells and Astrocytes, a Comparative Review.” Neurochemical Research 46, no. 10: 2525–2537. 33523395 10.1007/s11064-021-03255-8

[glia70190-bib-0024] Heinonen, E. , and R. Lammintausta . 1991. “A Review of the Pharmacology of Selegiline.” Acta Neurologica Scandinavica 84, no. S136: 44–59. 10.1111/j.1600-0404.1991.tb05020.x 1686954

[glia70190-bib-0025] Hernández‐Reyes, J. E. , A. B. Salinas‐Abarca , G. C. Vidal‐Cantú , et al. 2019. “ *α*5 GABA_A_ Receptors Play a Pronociceptive Role and Avoid the Rate‐Dependent Depression of the Hoffmann Reflex in Diabetic Neuropathic Pain and Reduce Primary Afferent Excitability.” Pain 160, no. 6: 1448–1458. 31107414 10.1097/j.pain.0000000000001515

[glia70190-bib-0026] Horton, R. , and B. Meldrum . 1973. “Seizures Induced by Allylglycine, 3‐Mercaptopropionic Acid and 4‐Deoxypyridoxine in Mice and Photosensitive Baboons, and Different Modes of Inhibition of Cerebral Glutamic Acid Decarboxylase.” British Journal of Pharmacology 49, no. 1: 52–63. 4207045 10.1111/j.1476-5381.1973.tb08267.x PMC1776427

[glia70190-bib-0027] Jo, S. , O. Yarishkin , Y. J. Hwang , et al. 2014. “GABA From Reactive Astrocytes Impairs Memory in Mouse Models of Alzheimer's Disease.” Nature Medicine 20, no. 8: 886–896. 10.1038/nm.3639 PMC8385452 24973918

[glia70190-bib-0028] Joo, J. , K. J. Kim , J. Lim , S. Y. Choi , W. Koh , and C. J. Lee . 2024. “Generation of Astrocyte‐Specific BEST1 Conditional Knockout Mouse With Reduced Tonic GABA Inhibition in the Brain.” Experimental Neurobiology 33, no. 4: 180–192. 39266474 10.5607/en24019 PMC11411089

[glia70190-bib-0029] Ju, Y. H. , J. Cho , J.‐Y. Park , et al. 2024. “Tonic Excitation by Astrocytic GABA Causes Neuropathic Pain by Augmenting Neuronal Activity and Glucose Metabolism.” Experimental & Molecular Medicine 56, no. 5: 1193–1205. 38760512 10.1038/s12276-024-01232-z PMC11148027

[glia70190-bib-0030] Kołosowska, K. A. , G. Schratt , and J. Winterer . 2023. “microRNA‐Dependent Regulation of Gene Expression in GABAergic Interneurons.” Frontiers in Cellular Neuroscience 17: 1188574. 37213213 10.3389/fncel.2023.1188574 PMC10196030

[glia70190-bib-0031] Kwak, H. , W. Koh , S. Kim , et al. 2020. “Astrocytes Control Sensory Acuity via Tonic Inhibition in the Thalamus.” Neuron 108, no. 4: 691–706. 32905785 10.1016/j.neuron.2020.08.013

[glia70190-bib-0032] Lee, H. Y. , J. M. Lee , H.‐L. Lee , et al. 2025. “Astrocytic Monoamine Oxidase B (MAOB)–Gamma‐Aminobutyric Acid (GABA) Axis as a Molecular Brake on Repair Following Spinal Cord Injury.” Signal Transduction and Targeted Therapy 10: 295. 40931022 10.1038/s41392-025-02398-2 PMC12423301

[glia70190-bib-0033] Lee, S. , B.‐E. Yoon , K. Berglund , et al. 2010. “Channel‐Mediated Tonic GABA Release From Glia.” Science 330, no. 6005: 790–796. 20929730 10.1126/science.1184334

[glia70190-bib-0034] Leitão, A. L. , and F. J. Enguita . 2022. “A Structural View of miRNA Biogenesis and Function.” Non‐Coding RNA 8, no. 1: 10. 35202084 10.3390/ncrna8010010 PMC8874510

[glia70190-bib-0035] Loeza‐Alcocer, E. , M. Canto‐Bustos , J. Aguilar , R. González‐Ramírez , R. Felix , and R. Delgado‐Lezama . 2013. “ *α*5GABA_A_ Receptors Mediate Primary Afferent Fiber Tonic Excitability in the Turtle Spinal Cord.” Journal of Neurophysiology 110, no. 9: 2175–2184. 23966669 10.1152/jn.00330.2013

[glia70190-bib-0036] Löscher, W. , D. Hönack , and M. Gramer . 1989. “Use of Inhibitors of *γ*‐Aminobutyric Acid (GABA) Transaminase for the Estimation of GABA Turnover in Various Brain Regions of Rats: A Reevaluation of Aminooxyacetic Acid.” Journal of Neurochemistry 53, no. 6: 1737–1750. 2809589 10.1111/j.1471-4159.1989.tb09239.x

[glia70190-bib-0037] Ma, K. , A. Xy , S. Cui , M.‐R. Sun , Y.‐C. Xue , and J.‐H. Wnag . 2016. “Impaired GABA Synthesis, Uptake and Release Are Associated With Depression‐Like Behaviors Induced by Chronic Mild Stress.” Translational Psychiatry 6: e910. 27701406 10.1038/tp.2016.181 PMC5315548

[glia70190-bib-0038] Manrique, C. , V. Compan , C. Rosselet , and S. G. D. Duflo . 2009. “Specific Knock‐Down of GAD67 in the Striatum Using Naked Small Interfering RNAs.” Journal of Biotechnology 142, no. 3–4: 185–192. 19497341 10.1016/j.jbiotec.2009.05.009

[glia70190-bib-0039] McGrath, A. P. , K. M. Hilmer , C. A. Collyer , et al. 2009. “Structure and Inhibition of Human Diamine Oxidase.” Biochemistry 48, no. 41: 9810–9822. 19764817 10.1021/bi9014192 PMC2791411

[glia70190-bib-0040] Minchin, M. 1975. “Factors Influencing the Efflux of [^3^H] Gammaaminobutyric Acid From Satellite Glial Cells in Rat Sensory Ganglia.” Journal of Neurochemistry 24, no. 3: 571–577. 234523 10.1111/j.1471-4159.1975.tb07676.x

[glia70190-bib-0041] Minchin, M. , and L. Iversen . 1974. “Release of [^3^H] Gamma‐Aminobutyric Acid From Glial Cells in Rat Dorsal Root Ganglia.” Journal of Neurochemistry 23, no. 3: 533–540. 4153611 10.1111/j.1471-4159.1974.tb06056.x

[glia70190-bib-0042] Mitchell, S. J. , and R. A. Silver . 2003. “Shunting Inhibition Modulates Neuronal Gain During Synaptic Excitation.” Neuron 38, no. 3: 433–445. 12741990 10.1016/s0896-6273(03)00200-9

[glia70190-bib-0043] Neal, M. , and N. Bowery . 1979. “Differential Effects of Veratridine and Potassium Depolarization on Neuronal and Glial GABA Release.” Brain Research 167, no. 2: 337–343. 445133 10.1016/0006-8993(79)90827-8

[glia70190-bib-0044] Oh, S.‐J. , and C. J. Lee . 2017. “Distribution and Function of the Bestrophin‐1 (Best1) Channel in the Brain.” Experimental Neurobiology 26, no. 3: 113–121. 28680296 10.5607/en.2017.26.3.113 PMC5491579

[glia70190-bib-0045] Orrego, F. 1980. “Criteria for the Identification of Central Neurotransmitters, and Their Application to Studies With Some Nerve Tissue Preparations in Vitro.” Commentaries in the Neurosciences: 161–181. 10.1016/0306-4522(79)90186-6 40157

[glia70190-bib-0046] Pegg, A. E. 2006. “Regulation of Ornithine Decarboxylase.” Journal of Biological Chemistry 281, no. 21: 14529–14532. 16459331 10.1074/jbc.R500031200

[glia70190-bib-0047] Perez‐Sanchez, J. , L. E. Lorenzo , I. Lecker , et al. 2017. “ *α*5 GABA_A_ Receptors Mediate Tonic Inhibition in the Spinal Cord Dorsal Horn and Contribute to the Resolution of Hyperalgesia.” Journal of Neuroscience Research 95, no. 6: 1307–1318. 27792253 10.1002/jnr.23981

[glia70190-bib-0048] Pillai, R. S. , S. N. Bhattacharyya , and W. Filipowicz . 2007. “Repression of Protein Synthesis by miRNAs: How Many Mechanisms?” Trends in Cell Biology 17, no. 3: 118–126. 17197185 10.1016/j.tcb.2006.12.007

[glia70190-bib-0049] Pineda‐Farias, J. B. , P. Barragán‐Iglesias , E. Loeza‐Alcocer , et al. 2015. “Role of Anoctamin‐1 and Bestrophin‐1 in Spinal Nerve Ligation‐Induced Neuropathic Pain in Rats.” Molecular Pain 11: s12990–s12991. 10.1186/s12990-015-0042-1 PMC4487556 26130088

[glia70190-bib-0050] Price, T. J. , F. Cervero , and Y. D. Koninck . 2005. “Role of Cation‐Chloride‐Cotransporters (CCC) in Pain and Hyperalgesia.” Current Topics in Medicinal Chemistry 5, no. 6: 547–555. 16022677 10.2174/1568026054367629 PMC1472095

[glia70190-bib-0051] Quiróz‐González, S. , R. E. Escartín‐Pérez , F. Paz‐Bermudez , et al. 2013. “Endogenous Content and Release of [3 H]‐GABA and [3 H]‐Glutamate in the Spinal Cord of Chronically Undernourished Rat.” Neurochemical Research 38: 23–31. 22983619 10.1007/s11064-012-0881-3

[glia70190-bib-0052] Raboni, S. , F. Spyrakis , B. Campanini , et al. 2010. “7.10‐Pyridoxal 5′‐Phosphate‐Dependent Enzymes: Catalysis, Conformation, and Genomics.” In Comprehensive Natural Products II, 273–350. Elsevier. vol. 7.

[glia70190-bib-0053] Rodríguez‐Palma, E. J. , Y. E. De la Luz‐Cuellar , A. M. Islas‐Espinoza , et al. 2023. “Activation of *α*6‐Containing GABA_A_ Receptors Induces Antinociception Under Physiological and Pathological Conditions.” Pain 164, no. 5: 948–966. 36001074 10.1097/j.pain.0000000000002763 PMC9950299

[glia70190-bib-0054] Schon, F. , and J. Kelly . 1974a. “Autoradiographic Localisation of [^3^H] GABA and [^3^H] Glutamate Over Satellite Glial Cells.” Brain Research 66, no. 2: 275–288.

[glia70190-bib-0055] Schon, F. , and J. Kelly . 1974b. “The Characterisation of [^3^H] GABA Uptake Into the Satellite Glial Cells of Rat Sensory Ganglia.” Brain Research 66, no. 2: 289–300.

[glia70190-bib-0056] Sellström, Å. , and A. Hamberger . 1976. “ *γ*‐Aminobutyric Acid Release From Neurons and Glia.” Acta Physiologica Scandinavica 98, no. 1: 94–102. 970161 10.1111/j.1748-1716.1976.tb10307.x

[glia70190-bib-0057] Vargas, O. , M. D. C. D. de Lorenzo , and F. Orrego . 1977. “Effect of Elevated Extracellular Potassium on the Release of Labelled Noradrenaline, Glutamate, Glycine, *β*‐Alanine and Other Amino Acids From Rat Brain Cortex Slices.” Neuroscience 2, no. 3: 383–390. 896045 10.1016/0306-4522(77)90004-5

[glia70190-bib-0058] Vargas‐Parada, A. , E. Loeza‐Alcocer , R. González‐Ramírez , et al. 2021. “ *γ*‐Aminobutyric Acid (GABA) From Satellite Glial Cells Tonically Depresses the Excitability of Primary Afferent Fibers.” Neuroscience Research 170: 50–58. 32987088 10.1016/j.neures.2020.08.007

[glia70190-bib-0059] Verkhratsky, A. , and H. Kettenmann . 1996. “Calcium signalling in glial cells.” Trends in Neurosciences 19, no. 8: 346–352. 8843604 10.1016/0166-2236(96)10048-5

[glia70190-bib-0060] Watanabe, M. , K. Maemura , K. Kanbara , T. Tamayama , and H. Hayasaki . 2002. “GABA and GABA Receptors in the Central Nervous System and Other Organs.” International Review of Cytology 213: 1–47. 11837891 10.1016/s0074-7696(02)13011-7

[glia70190-bib-0061] Witschi, R. , P. Punnakkal , J. Paul , et al. 2011. “Presynaptic *α*2‐ GABA_A_ Receptors in Primary Afferent Depolarization and Spinal Pain Control.” Journal of Neuroscience 31, no. 22: 8134–8142. 21632935 10.1523/JNEUROSCI.6328-10.2011 PMC3567284

[glia70190-bib-0062] Woo, J. , J. O. Min , D.‐S. Kang , et al. 2018. “Control of Motor Coordination by Astrocytic Tonic GABA Release Through Modulation of Excitation/Inhibition Balance in Cerebellum.” Proceedings of the National Academy of Sciences of the United States of America 115, no. 19: 5004–5009. 29691318 10.1073/pnas.1721187115 PMC5948981

[glia70190-bib-0063] Yoon, B. E. , J. Woo , Y. E. Chun , et al. 2014. “Glial GABA, Synthesized by Monoamine Oxidase B, Mediates Tonic Inhibition.” Journal of Physiology 592, no. 22: 4951–4968. 25239459 10.1113/jphysiol.2014.278754 PMC4259537

[glia70190-bib-0064] Yu, X.‐J. , T. Xiao , X.‐J. Liu , et al. 2021. “Effects of Nrf1 in Hypothalamic Paraventricular Nucleus on Regulating the Blood Pressure During Hypertension.” Frontiers in Neuroscience 15: 805070. 34938159 10.3389/fnins.2021.805070 PMC8685333

