# Supplementary material for: Satellite Glial Cells Synthesize and Release GABA to Activate Extrasynaptic GABAA Receptors That Modulate Dorsal Root Ganglia Neuron Excitability
Source: Glia. 2026 Jul 20;74(9):e70190. doi: 10.1002/glia.70190 (PMC13385656; doi:10.1002/glia.70190)
Supplement: Supplementary file 1 — Table S1: Sequences of oligonucleotides RT‐PCR. [file GLIA-74-0-s002.docx]

| Table 1. Sequences of oligonucleotides RT-PCR | | | | |
| --- | --- | --- | --- | --- |
| **Protein** | **Gen** | **Sense** | **Antisense** | **bp** |
| MAOB | *maob* | ATGAGCAACAAATGCGATGT | CTCAACTTCATTCACTTTATAGGTC | 258 |
| DAO | *aoc1* | ATGCGGGTTTGAAGGGATAT | GATTGGTGAGGTTTTCCAGC | 300 |
| ODC | *odc1* | ATGGGCAGCTTTACTAAGGA | AATCAAATCCTGTCCCAATG | 265 |
| Best1 | *vmd2* | ATGACTATCACCTACACAAAC | GTAACATAGAAACCCAGAAC | 260 |
| GAD_65_ | *gad2* | GCATCTCCGGGCTCTGGCTTTT | ATCTCCTTTGGGGCAGCTGCAG | 255 |
| GAD_67_ | *gad1* | CATCTTCCACGCCTTCGCCT | GGGCACCAGGGTCACTGTTT | 252 |
| vGAT | *Slc32a1* | ACATTCATTATCAGCGCGGC | ATCTTGCCGGTGTAGCAGCA | 250 |
