# Supplementary material for: Satellite Glial Cells Synthesize and Release GABA to Activate Extrasynaptic GABAA Receptors That Modulate Dorsal Root Ganglia Neuron Excitability
Source: Glia. 2026 Jul 20;74(9):e70190. doi: 10.1002/glia.70190 (PMC13385656; doi:10.1002/glia.70190)
Supplement: Supplementary file 2 — Table S2: Antibodies for immunofluorescence and western blot. [file GLIA-74-0-s001.docx]

| Table 2. Antibodies for Immunofluorescence and Western Blot | | | |
| --- | --- | --- | --- |
| **Antibody** | **Host** | **Company** **(#Catalog)** | **Dilution** |
| Monoamine oxidase-B | Mouse monoclonal | Santacruz (sc-515354) | 1:800 |
| Diamine oxidase | Mouse monoclonal | Santacruz (sc-515908) | 1:100 |
| Ornithine decarboxylase | Mouse monoclonal | Abcam (ab66067) | 1:100 |
| Bestrophin-1 | Rabbit polyclonal | Alomone (ABC-001) | WB 1:300 |
| Bestrophin-1 | Rabbit polyclonal | Abcam (AB14927) | 1:100 |
| GABA | Mouse monoclonal | Abcam (ab86186) | 1:100 |
| GAD65+67 | Rabbit monoclonal | Abcam (ab183999) | IF 1:50  WB 1:1000 |
| VGAT | Rabbit polyclonal | Thermo Fisher (PA5-27569) | WB 1:1000 |
| VGAT | Mouse monoclonal | Santacruz (sc-393373) | 1:200 |
| GAT3 | Mouse monoclonal | Santacruz (sc-376001) | 1:250 |
| GABA-T | Mouse monoclonal | Santacruz (sc-393769) | 1:200 |
| NeuN | Rabbit monoclonal | Cell Signaling Technology (D3S3I) | 1:500 |
| NeuN | Mouse monoclonal | Thermo Fisher (MA5-33103) | 1:100 |
| Glutamine synthetase | Rabbit polyclonal | Abcam (ab73593) | 1:200 |
| Glutamine synthetase | Mouse monoclonal | Millipore (MAB302) | 1:200 |
| Actin | Mouse | Genetex | WB 1:10000 |
| Donkey anti-rabbit Alexa-488 | Donkey polyclonal | Jackson InmunoResearch (711-545-152) | 1:200 |
| Donkey anti-mouse Alexa-594 | Donkey polyclonal | Jackson InmunoResearch (715-585-150) | 1:200 |
| Anti-mouse horseradish peroxidase (HRP)-coupled secondary antibody | - | Jackson InmunoResearch (115-035-003) | WB 1:5000 |
| Anti-rabbit horseradish peroxidase (HRP)-coupled secondary antibody | - | Jackson InmunoResearch (111-035-003) | WB 1:5000 |
| Hoechst | - | Invitrogen (H1398) | 1:2000 |

IF: Immunofluorescence; WB: Western blot
